# A Focused Narrative Review of Selected Evidence on Gymnastics Training Optimization, Technology, Athlete Health, and Injury Prevention

**DOI:** 10.3390/healthcare14193285

**Published:** 2026-10-02

**Authors:** Nicoleta Zaharie, Sonia Gabriela Neagu, Delia Badescu, Vlad Teodor Grosu, Alina Georgeta Mag, Cristian Stanciu, Ioan Sabin Sopa

**Affiliations:** 1 Department of Environmental Science, Physics, Physical Education and Sport, Faculty of Science, Lucian Blaga University of Sibiu, 550012 Sibiu, Romania; nicoleta.zaharie@ulbsibiu.ro (N.Z.); soniagabriela.neagu@ulbsibiu.ro (S.G.N.); cristiandumitru.stanciu@ulbsibiu.ro (C.S.); 2 Department of Mechatronics and Machine Dynamics, Faculty of Automotive, Mechatronics and Mechanical Engineering, Technical University of Cluj-Napoca, 400461 Cluj-Napoca, Romania; vlad.grosu@mdm.utcluj.ro; 3 Department of Teacher Training, Faculty of Social Sciences and Humanities, Lucian Blaga University of Sibiu, 550324 Sibiu, Romania; alina.mag@ulbsibiu.ro

**Keywords:** gymnastics, sustainable training, injury prevention, athlete health, training load, sports technology, biomechanical assessment, long-term athlete development

## Abstract

**Highlights:**

**What are the main findings?**

- Sustainable gymnastics training depends on balancing performance development with injury prevention, appropriate load management, and the protection of athletes’ physical and psychological health.
- Emerging technologies, including wearable sensors, video analysis, and artificial intelligence, show potential to support movement assessment, workload monitoring, and the identification of variables relevant to athlete health and training safety; however, evidence for several applications remains preliminary and requires further gymnastics-specific validation.

**What are the implications of the main findings?**

- Coaches may integrate age-appropriate training, progressive load management, recovery strategies, and regular health monitoring into long-term gymnastics programmes.
- Combining evidence-informed coaching with accessible monitoring technologies may support training decisions, athlete monitoring, and long-term participation and development.

**Abstract:**

**Background:** Sustainable gymnastics training requires a balance between performance development, injury prevention, workload management, athlete health, psychological well-being, and long-term participation. This focused narrative review aimed to synthesize selected evidence on training optimization, technological applications, athlete health and injury prevention in gymnastics. **Methods:** A structured narrative review was conducted using the Web of Science Core Collection. The literature search covered publications issued between 1 January 2005 and 30 September 2025, with the final search performed on 30 September 2025. English-language publications retrieved through three predefined Topic searches in the Web of Science Core Collection were considered. The searches covered the period from 1 January 2005 to 30 September 2025 and were restricted to the Sport Sciences category. No additional publications were identified through reference-list screening. Three complementary thematic searches addressing gymnastics training models, technological applications, and injury prevention identified 485 records. After applying the eligibility criteria, removing duplicates, assessing full texts, and excluding ineligible or retracted publications, 140 publications were included in the narrative synthesis. No formal risk-of-bias assessment was performed. **Results:** The reviewed evidence emphasized progressive and individualized training, workload monitoring, recovery, physical preparation, age-appropriate development, and early identification of pain, fatigue, and other health-related concerns. Injury occurrence was associated with biomechanical loading, rapid growth, training exposure, previous injuries, insufficient recovery, menstrual dysfunction, psychological factors, and discipline-specific technical demands. Wearable sensors, video analysis, biomechanical modelling, artificial intelligence, virtual reality, and digital learning platforms supported movement assessment, technical feedback, training monitoring, and education. Evidence for several emerging technological applications, particularly artificial intelligence and automated assessment, remains preliminary and requires further validation in gymnastics-specific and real-world settings. However, substantial differences in study designs, populations, technologies, outcome measures, and methodological characteristics limited direct comparison across studies. Coaching communication, athlete motivation, psychological well-being, and access to validated technologies also influenced the sustainability of gymnastics training. **Conclusions:** Sustainable gymnastics training may benefit from an interdisciplinary and athlete-centered approach integrating individualized load management, injury prevention, health monitoring, recovery, educational coaching practices, and appropriately validated technologies. The evidence identified in this review highlights converging themes suggesting the potential value of combining these components to support safe performance development and long-term participation. However, more longitudinal, discipline-specific, and methodologically consistent research is needed.

## 1. Introduction

Modern sports training increasingly emphasizes sustainable approaches that support long-term athlete development, injury prevention, performance optimization, and the responsible use of scientific methods and technologies. In gymnastics, where technical precision and high physical demands coexist from the early stages of participation, these principles are particularly relevant for protecting athletes’ physical and psychological health while supporting performance development.

In this review, the term “gymnastics” is used in a broad operational sense. It includes recognized competitive disciplines, such as artistic gymnastics, rhythmic gymnastics, trampoline gymnastics, acrobatic gymnastics, aerobic gymnastics, and parkour, as well as non-competitive participation contexts, including recreational, general, school, and university gymnastics. Adapted, therapeutic, and corrective gymnastics are included as educational or health-related applications of gymnastics-based movement rather than as competitive disciplines. When evidence refers to a specific discipline, participation context, or therapeutic application, this distinction is stated explicitly.

The sustainable development of gymnastics depends on effective interaction between research, education, coaching practice, and technological innovation. This integration can support evidence-informed decision making and improve the quality and efficiency of training [1]. Because postural control and movement accuracy are fundamental to gymnastics performance, technologies for human posture recognition and movement analysis have become increasingly relevant. These systems can assist in monitoring technical execution, identifying movement errors, and providing objective information that complements coaches’ observations [2,3,4].

Kinematic monitoring, wearable sensors, and automated data analysis provide further opportunities for individualized assessment and technical correction. The combination of verbal, visual, and sensory feedback can facilitate motor learning, movement control, and adaptation to different training demands [5,6,7,8,9]. Such technologies are most valuable when they complement coaching expertise and contribute to informed decisions based on the characteristics and needs of individual athletes.

Physical preparation remains fundamental to gymnastics performance. Functional and neuromuscular training can improve explosive strength, jumping ability, postural control, and other physical capacities relevant to gymnastics performance and safe technical execution [10,11,12,13,14,15,16]. However, these performance-related outcomes should not be interpreted as direct evidence of reduced injury incidence. Grip strength development, isometric interventions, and individualized training strategies may further improve coordination, flight time, and gymnastics-specific technical performance [17,18,19,20]. These findings reinforce the importance of adapting training content and load to the athlete’s developmental stage and performance requirements. Broader theoretical approaches to physical fitness programming through aerobics were also consulted to contextualize the organization and adaptation of exercise programmes [21].

Effective athlete development may also integrate psychological, social, and ethical considerations. Research involving junior sports acrobatics has examined relationships between athletes’ personality traits and sport classifications [22]. Research in elite women’s gymnastics has also shown how organizational expectations and performance structures can shape parental involvement and the socialization of parents within the sporting environment [23]. Gymnastics further reflects broader social and cultural dimensions, including perceptions of femininity and masculinity within competitive structures [24]. Sustainable training therefore extends beyond physical performance and includes athlete welfare, social relationships, parental involvement, and the quality of the sporting environment.

Rapid technological development has expanded the use of Internet of Things systems, sensor-based artificial intelligence, and automated performance analysis in sport. In gymnastics, these tools may support performance monitoring, talent identification, technical evaluation, and more objective assessment [25,26]. Automated judging systems have been investigated as potential methods for supporting the consistency and objectivity of performance evaluation. Direct evidence from gymnastics includes sensor-based jump classification and computerized judging-support systems [27,28]. Related studies from physical education and other judged sports provide indirect evidence concerning diagnostic accuracy, video-based measurement, and artificial intelligence in performance evaluation [29,30,31]. The transferability of this indirect evidence to gymnastics requires discipline-specific validation.

Technological and methodological changes also influence gymnastics education. Digital learning environments, personalized instruction, student-centered approaches, and the Flipped Classroom model provide alternative ways of teaching motor skills [32]. Educational approaches that combine video feedback, structured movement activities, and interactive technologies may support motor learning, self-assessment, motivation, and student engagement in gymnastics and physical education [33,34]. National gymnastics programmes have similarly been used to encourage organized physical activity and promote lifelong participation [35,36]. A broader theoretical perspective on artificial intelligence in education was also consulted to contextualize the potential contribution of emerging technologies to teaching, learning, and human–machine collaboration [37].

The relevance of gymnastics is not limited to competitive sport. Recreational gymnastics may contribute to maintaining functional capacity and reducing the risk of traumatic falls among older adults. Corrective gymnastics and therapeutic swimming programmes may also support motor development and the management of musculoskeletal deviations in children, including those with Down syndrome [38,39,40,41]. These applications illustrate the potential contribution of gymnastics across different ages, ability levels, and health-related settings.

Recent literature has placed greater emphasis on sustainable training and injury prevention. Because gymnastics combines repetitive loading, complex technical skills, high movement amplitudes, and substantial biomechanical demands, athletes may be exposed to both acute and overuse injuries [42,43,44,45]. Identifying modifiable risk factors and monitoring training-related variables are therefore central to prevention. Flexibility training and stretching can influence range of motion and acute performance responses [46,47]. However, the available evidence does not establish that stretching alone reduces the incidence of gymnastics injuries.

Injury prevention may benefit from coordinated involvement from coaches, athletes, sports scientists, medical professionals, and families. Systematic monitoring and athlete self-monitoring can facilitate the early identification of pain, fatigue, and functional limitations, allowing timely modification of training loads and preventive interventions [48,49,50,51,52,53,54,55]. This is particularly important in young gymnasts, whose physical development and response to training may change considerably throughout the stages of maturation.

Despite the growing volume of gymnastics research, the literature remains fragmented. Studies frequently address isolated components of training and differ substantially in research design, populations, gymnastics disciplines, participation contexts, educational or health-related applications, assessment methods, and outcome measures. This heterogeneity limits direct comparison and makes it difficult to translate individual findings into a coherent organizing perspective for training practice. At the same time, technological developments, evolving training methods, and increased attention to athlete health have generated new areas of research that require broader synthesis.

Previous literature has addressed specific components of athlete preparation and health, including technological applications in sport and gymnastics [1,25,26,27,28,29,30,31], physical and neuromuscular preparation [10,11,12,13,14,15,16,17,18,19,20], injury-related factors and athlete monitoring [43,44,45,46,47,48,49,50,51,52,53,54,55], and growth- and health-related outcomes relevant to gymnastics participation [56,57,58,59,60,61,62,63,64,65]. However, these areas have generally been investigated separately across different gymnastics disciplines, populations, participation contexts, and methodological approaches. This predominantly domain-specific evidence base highlights the value of examining the interconnections and converging themes across these areas from a broader sustainability-oriented perspective.

Against this background, important questions remain regarding training efficiency, athlete safety, exercise dosage, long-term development, and the appropriate integration of emerging technologies into coaching and educational practice [66,67,68,69,70,71].

Accordingly, this focused narrative review aims to synthesize selected evidence identified through three predefined exact-phrase searches in the Web of Science Core Collection. The review examines findings related to gymnastics training optimization, technological applications, and athlete health and injury prevention within the retrieved corpus. It does not aim to provide an exhaustive mapping of the multidisciplinary gymnastics literature or to establish clinical or professional practice guidelines.

Building on the predominantly topic- and discipline-specific focus of previous literature, the present review adopts an integrated perspective linking physical, psychological, social and organizational, technological, and performance-related dimensions from a sustainability-oriented perspective. Physical sustainability refers to the management of training load, injury risk, recovery, and athlete health through practices adapted to age, developmental stage, health status, and performance level. Psychological sustainability refers to motivation, emotional well-being, perceived support, autonomy, and continued engagement in gymnastics. Social and organizational sustainability refers to athlete safeguarding, supportive relationships, coaching education, equitable access, and stable training structures. Technological sustainability refers to the appropriate, accessible, and evidence-informed use of technologies, with consideration of measurement validity, practical value, data protection, resource requirements, and long-term feasibility.

Performance sustainability refers to the capacity to support athletic development and technical progression while protecting health, psychological well-being, education, and long-term participation. In this review, psychological and social factors are treated as distinct but interacting dimensions. Therefore, the broader term “psychosocial” is used only when a cited publication addresses both dimensions together. Through this broader perspective, the review aims to provide relevant evidence for coaches, physical education teachers, health professionals, and researchers working across recreational, educational, compensatory, and performance-oriented gymnastics.

## 2. Materials and Methods

### 2.1. Review Design

This study was conducted as a structured narrative review using a focused, predefined literature search strategy, concerning modern training methodologies, technological applications, athlete health, and injury prevention in gymnastics. The review combined a structured literature search with descriptive analysis and thematic narrative synthesis.

The use of structured procedures for literature identification, eligibility assessment, independent screening, data extraction, and study selection was intended to enhance transparency and reproducibility rather than to define the review as a systematic or scoping review. The review was not designed to provide an exhaustive mapping of the gymnastics literature; rather, it used focused thematic searches to identify evidence relevant to three predefined domains and integrated this heterogeneous evidence through descriptive analysis and thematic narrative synthesis. Accordingly, the term “structured narrative review” is used throughout the manuscript to reflect both the structured review procedures and the narrative, interpretative nature of the evidence synthesis.

A narrative approach was selected because the literature covered different gymnastics disciplines, participation contexts, educational or health-related applications, participant groups, competitive levels, research designs, interventions, technologies, and outcome measures. This methodological heterogeneity did not permit a valid statistical aggregation of the findings.

The review addressed three objectives within the identified evidence corpus:

1. To summarize selected evidence on gymnastics training methods related to physical preparation, technical development, and athlete participation.
2. To examine selected applications of biomechanical analysis, wearable sensors, video systems, artificial intelligence, and other technologies in gymnastics training, education, monitoring, and assessment.
3. To describe injury patterns, associated factors, preventive interventions, workload management, recovery, and health-monitoring practices reported in the included gymnastics literature.

Publication year was summarized descriptively for the complete corpus. Country or institutional affiliation, gymnastics discipline or participation context, participant age, and training level were extracted at the publication level and reported in Supplementary Table S1. These variables were not treated as separate review objectives or subjected to inferential analysis.

### 2.2. Information Source

The Web of Science Core Collection was used as the sole bibliographic information source. The Sport Sciences category was applied to increase the specificity of the searches and identify literature directly related to gymnastics and sport practice. No separate searches were conducted in medical, health-related, educational, biomechanical, engineering, or technological categories. Records classified under Sport Sciences could also have additional Web of Science category assignments. The review covered publications issued between 1 January 2005 and 30 September 2025. The final database search was conducted on 30 September 2025.

The period from 1 January 2005 to 30 September 2025 was predefined to focus the review on contemporary evidence and developments from the most recent two decades relevant to modern gymnastics training, technological applications, and current approaches to athlete health and injury prevention. The year 2005 was therefore used as a pragmatic temporal boundary rather than as a threshold marking the emergence of these research areas. We acknowledge that important foundational research on gymnastics training, injury epidemiology, maturation, bone health, and biomechanics predates this period and was consequently outside the predefined search window.

### 2.3. Search Strategy

Three separate, predefined thematic searches were conducted using the Web of Science Topic field (TS), which covers the title, abstract, author keywords, and Keywords Plus.

The following search expressions were entered separately:

“gymnastics training models”

“technology in gymnastics”

“injury prevention in gymnastics training”

These queries corresponded to the three predefined areas of the review: gymnastics training methods, technological applications in gymnastics, and athlete health and injury prevention. The searches were restricted by publication period, English language, and the Web of Science Sport Sciences category. No separate searches were conducted in other Web of Science categories, and no additional publications were identified or included through reference-list screening. All publications included in the final corpus were retrieved through the three database searches and were available for full-text assessment.

After applying the publication period, language, and Web of Science category filters, the three searches identified 485 records: 197 records for gymnastics training models, 194 records for technology in gymnastics, and 94 records for injury prevention in gymnastics training.

The search results were exported from the Web of Science Core Collection as Microsoft Excel files. The exported information included the title, authors, publication year, source title, abstract, author keywords, Keywords Plus, document type, and DOI, when available. The files obtained from the three searches were combined into one Microsoft Excel dataset. Duplicate records were identified primarily by DOI. When a DOI was unavailable, the title, authors, publication year, and source title were compared. Two reviewers independently assessed the combined dataset for duplicate records. Disagreements were resolved through discussion and consensus.

The three searches were designed as focused thematic searches rather than as an exhaustive systematic search of the entire gymnastics literature. Exact phrases were used to increase specificity and to identify publications directly connected with the three predefined review domains. This approach reduced the retrieval of publications with only a peripheral connection to gymnastics training. However, the use of highly specific phrases may have omitted relevant studies that used alternative terms, synonyms, discipline-specific terminology, or different descriptions of training, technology, athlete health, and injury prevention. Accordingly, the retrieved corpus should be considered a focused sample of contemporary literature relevant to the predefined review domains rather than a comprehensive representation of all gymnastics research.

The complete search strings, including the Web of Science field operator (TS), are reported below and in Table 1 to ensure reproducibility:

### 2.4. Eligibility Criteria

Publications were considered eligible when they met the following inclusion criteria:

1. They were retrieved through one of the three predefined Web of Science Core Collection searches conducted using the Sport Sciences category. They were published in English.
2. They were published between 1 January 2005 and 30 September 2025.
3. They examined at least one of the three main areas of the review: gymnastics training optimization, technological applications in gymnastics, or athlete health and injury prevention.
4. They provided empirical, methodological, theoretical, or review-based evidence relevant to gymnastics training, education, performance, athlete health, or injury prevention.

The broad eligibility criteria were intentionally selected to capture the multidisciplinary and methodologically heterogeneous evidence relevant to the three predefined review domains. Different publication types were subsequently differentiated according to their methodological design and role in the narrative synthesis, as described in Section 2.7.

No document-type filter was applied during the database searches. During screening, full research articles, review articles, and full conference proceedings papers were eligible when they provided sufficient empirical, methodological, or conceptual information relevant to the objectives of the review. Editorials, conference abstracts, news items, commentaries, and publications without sufficient scientific content were excluded.

Publications were excluded when they met one or more of the following criteria:

1. Publications were considered applicable when they reported an empirical finding, assessment method, intervention, technological procedure, or conceptual contribution that could be linked explicitly to at least one of the three predefined review domains. Gymnastics-specific publications were required to examine a recognized gymnastics discipline, a clearly identified gymnastics participation context, or a gymnastics-based educational or health-related application. Publications involving adjacent sports, general physical education, or generic technological methods were retained only when their relevance to a specific review question could be stated explicitly. Such evidence was classified as indirect and was not interpreted as direct evidence of effectiveness in gymnastics.
2. They did not provide sufficient methodological or conceptual information to support data extraction and narrative synthesis.
3. They were editorials, conference abstracts, news items, commentaries, or other publications without sufficient scientific content.
4. They were duplicate records.
5. They had been withdrawn or retracted.

Two books were identified separately and used exclusively to provide theoretical context. They were not retrieved through the database searches and were not included among the 140 publications selected for the narrative synthesis.

### 2.5. Study Selection

Following export and dataset consolidation, seven duplicate records were identified and removed, leaving 478 records for title and abstract screening. Two authors independently assessed the titles and abstracts according to the predefined eligibility criteria. At this stage, 325 records were excluded because they did not demonstrate a sufficiently direct connection with gymnastics or with at least one of the three thematic domains of the review.

Sufficient thematic relevance was defined as the presence of extractable information concerning the population or context, study design or methodological approach, intervention or assessment procedure, and at least one outcome or conceptual contribution related to a predefined review domain. Publications were excluded at full-text assessment when gymnastics was mentioned only incidentally, when gymnastics-related findings could not be separated from broader results, or when the publication did not provide sufficient information to support data extraction and narrative interpretation.

Full texts were successfully retrieved for all 153 publications selected for eligibility assessment. Therefore, no publication reaching the full-text assessment stage was excluded because of lack of full-text accessibility. The full texts were assessed independently by the same two authors. Twelve publications were excluded because they did not meet the operational definition of sufficient thematic relevance, and one retracted publication was excluded.

**Figure 1:**
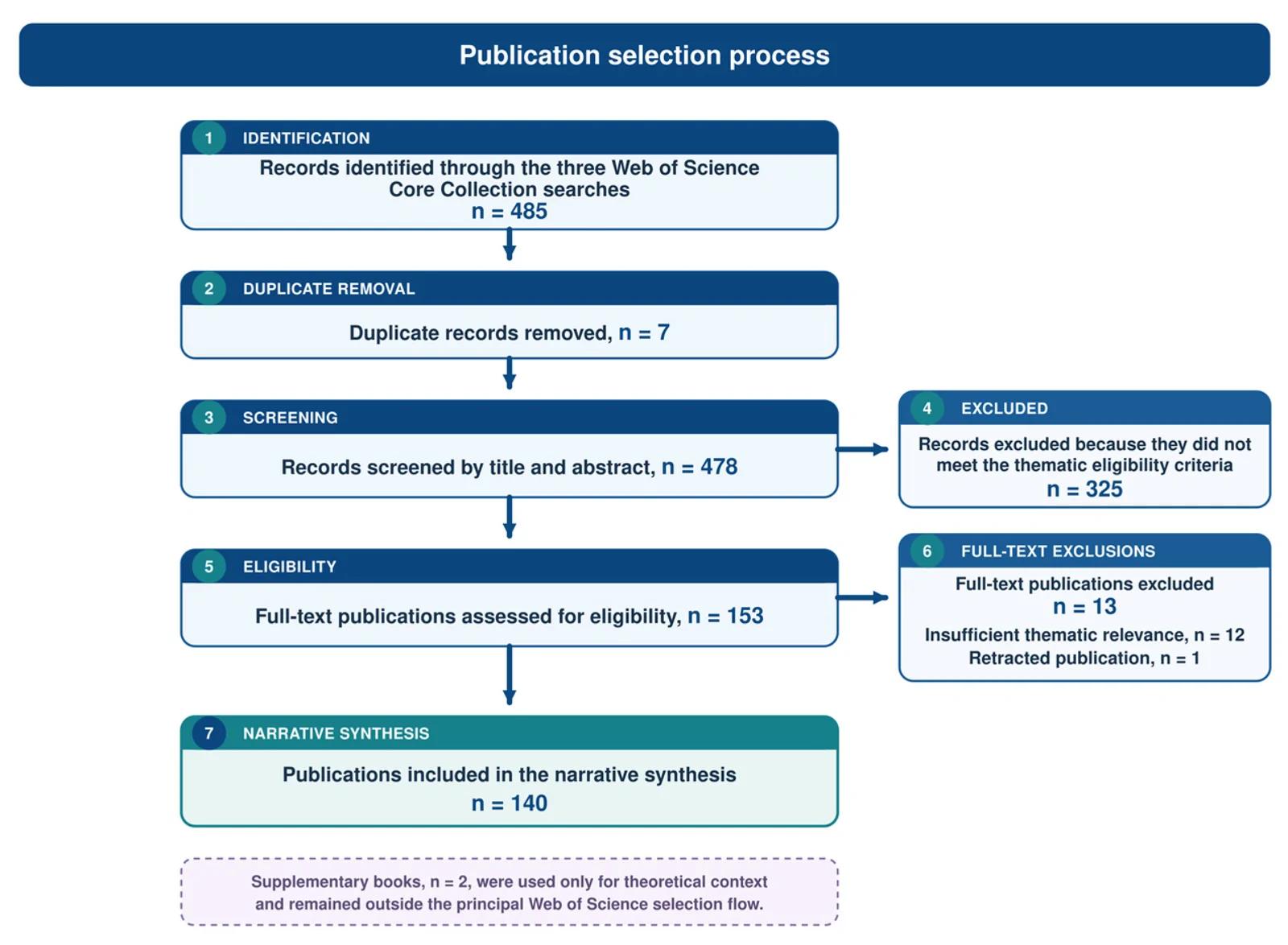
Flow diagram of publication identification, duplicate removal, title and abstract screening, full-text eligibility assessment, and inclusion in the narrative synthesis.

Consequently, 140 publications were included in the final narrative synthesis. The complete publication-selection process is presented in Figure 1.

### 2.6. Data Extraction

One author extracted the data using a predefined form, and a second author verified the extracted information against the full texts. Information was extracted from the included publications using a structured data-extraction framework. The following variables were considered:

- Author and year of publication.
- Country or institutional affiliation.
- Study design.
- Gymnastics discipline or activity area, participation context, and educational or health-related application.
- Participant characteristics.
- Participant age, competitive level, and training level.
- Biological maturation or developmental status, when explicitly reported.
- Sample size, when applicable.
- Training method, intervention, technology, or assessment procedure.
- Duration and frequency of the intervention, when applicable.
- Physical, technical, psychological, educational, technological, or health-related outcomes.
- Injury definition, injury location, mechanism, severity, or risk factor, when reported.
- Main findings.
- Practical implications.
- Methodological limitations reported by the publication authors.

Each publication was also classified according to the directness of its evidence. Gymnastics-specific evidence included studies conducted in recognized gymnastics disciplines, clearly defined gymnastics participation contexts, or explicit gymnastics-based educational or health-related applications. Adjacent evidence included studies from related sports, judged activities, physical education, or exercise settings that addressed a method, mechanism, or technology potentially relevant to gymnastics. Generic contextual evidence included broader methodological or theoretical publications without a gymnastics-specific population or setting. Adjacent and generic evidence was treated as indirect and was not used alone to support gymnastics-specific claims.

Gymnastics-related activities were coded using three separate dimensions: recognized gymnastics disciplines or activity areas, participation contexts, and educational or health-related applications. The recognized disciplines and activity areas represented in the review included men’s and women’s artistic gymnastics, rhythmic gymnastics, trampoline gymnastics, acrobatic gymnastics, aerobic gymnastics, parkour, and Gymnastics for All. The term “general gymnastics” was coded as Gymnastics for All only when the original publication used it in an equivalent sense. Participation contexts included competitive, recreational, school, and university settings. Adapted, therapeutic, and corrective gymnastics were coded as educational or health-related applications rather than as recognized sport disciplines.

Participant characteristics were coded according to chronological age, competitive level, training level, and type of participation. Biological maturation or developmental status was not treated as a mandatory data field because it was not reported consistently across the included publications. This information was recorded when explicitly reported by the original publication and was considered in the narrative synthesis. Biological maturation was not inferred from chronological age.

Detailed characteristics of the included publications, including retrieval query, evidence directness classification, country or institutional affiliation, gymnastics discipline or activity area, participation context or application, participant characteristics, study design, main topic, principal findings, and reported limitations, are presented in Supplementary Table S1.

### 2.7. Methodological Classification and Interpretation of Evidence

The included publications were classified according to their methodological design. The main categories were experimental studies, quasi-experimental studies, observational studies, cross-sectional studies, longitudinal studies, biomechanical investigations, technological validation studies, qualitative studies, reviews, and conceptual or educational studies.

The interpretation considered the methodological characteristics of each publication, including sample size, study design, duration, measurement procedures, control conditions, and applicability to real training environments.

Experimental and longitudinal findings received greater interpretative weight when the procedures and outcomes were clearly reported. Cross-sectional, descriptive, qualitative, and exploratory studies were used primarily to identify associations, experiences, technological possibilities, or areas requiring further investigation.

The review did not calculate an overall methodological quality score because it included highly heterogeneous research designs. The absence of a formal risk-of-bias assessment was considered when formulating the conclusions. Findings from small, uncontrolled, exploratory, or laboratory-based studies were not interpreted as evidence of causality or universal effectiveness.

Reviews and conceptual or educational publications were used primarily to provide contextual, integrative, or theoretical perspectives and were not assigned the same interpretative weight as primary empirical evidence. When conclusions reported in review articles overlapped with findings from primary studies included in the corpus, the reviews were used for contextual synthesis rather than treated as additional independent evidence supporting the same finding. This approach was used to minimize the risk of double-counting evidence represented both in primary studies and in included reviews.

### 2.8. Data Synthesis

A thematic narrative synthesis was performed because the studies differed substantially in their participants, interventions, gymnastics disciplines, measurement procedures, technologies, and outcomes.

The synthesis followed four stages:

1. The publications were classified according to the three primary review areas.
2. The main study characteristics and findings were extracted.
3. Similar findings were grouped into thematic categories.
4. Agreements, differences, methodological limitations, and practical implications were compared across the publications.

The first thematic area included training planning, physical preparation, technical learning, coordination, psychological preparation, athlete selection, exercise capacity, functional adaptations, educational gymnastics, and adapted gymnastics.

The second area included biomechanics, kinematic analysis, motion capture, wearable sensors, inertial measurement units, video analysis, digital learning systems, artificial intelligence, machine learning, automated movement recognition, and technology-assisted assessment.

The third area included injury prevalence, anatomical location, acute and overuse injuries, injury mechanisms, training exposure, workload, growth and maturation, previous injury, landing mechanics, pain monitoring, recovery, return to sport, and preventive exercise programmes.

The synthesis distinguished between findings directly reported by the included publications and broader interpretations formulated by the review authors. Causal language was avoided when the supporting evidence came from observational, cross-sectional, or exploratory studies. In such cases, findings were expressed using cautious terms such as “may”, “is associated with”, “suggests”, or “has the potential to”, according to the level and design of the available evidence.

### 2.9. Descriptive Analysis of the Literature

The included publications were analysed descriptively according to publication year, publication period, and principal thematic area. Frequencies were summarized using absolute values and percentages. Each publication was assigned to one principal thematic area according to its predominant focus to prevent double counting in the reported thematic frequencies. Additional publication-level characteristics, including country or institutional affiliation, gymnastics discipline, participation context or application, participant characteristics, participant age, training level, study design, main topic, principal findings, and reported limitations, were extracted and are presented in Supplementary Table S1. These additional characteristics were not subjected to separate quantitative or inferential analysis.

To provide a more informative mapping of the evidence base, the included publications were also cross-tabulated according to their principal review domain and methodological design. For this descriptive evidence mapping, each publication was assigned to one principal methodological design category: experimental/quasi-experimental, longitudinal, cross-sectional/observational, biomechanical/validation, qualitative, or review/conceptual. Each publication was counted once within its principal review domain and principal methodological design category to avoid double counting.

Publication year was assigned according to the final bibliographic year reported in the Web of Science record and used in the reference list. Early-access dates were not counted separately from the final publication year.

### 2.10. Review Workflow and Synthesis Structure

The review was reported as a structured narrative synthesis, with emphasis on transparency in the literature identification, study selection, data extraction, descriptive characterization, and thematic interpretation. The identification, screening, eligibility assessment, and final inclusion of publications are presented in the study selection flow diagram (Figure 1).

The review process was organized as a five-stage workflow comprising literature searching, eligibility assessment, data extraction, descriptive analysis, and thematic narrative synthesis. Within the final stage, the thematic narrative synthesis was conducted according to the four-step procedure described in Section 2.8. The thematic synthesis was structured around three main domains identified in relation to sustainable gymnastics training: training optimization, technological applications, and athlete health and injury prevention. This synthesis structure was used to organize heterogeneous evidence from different gymnastics disciplines, participation contexts, educational or health-related applications, populations, study designs, and methodological approaches while maintaining a consistent structure for interpretation.

**Figure 2:**
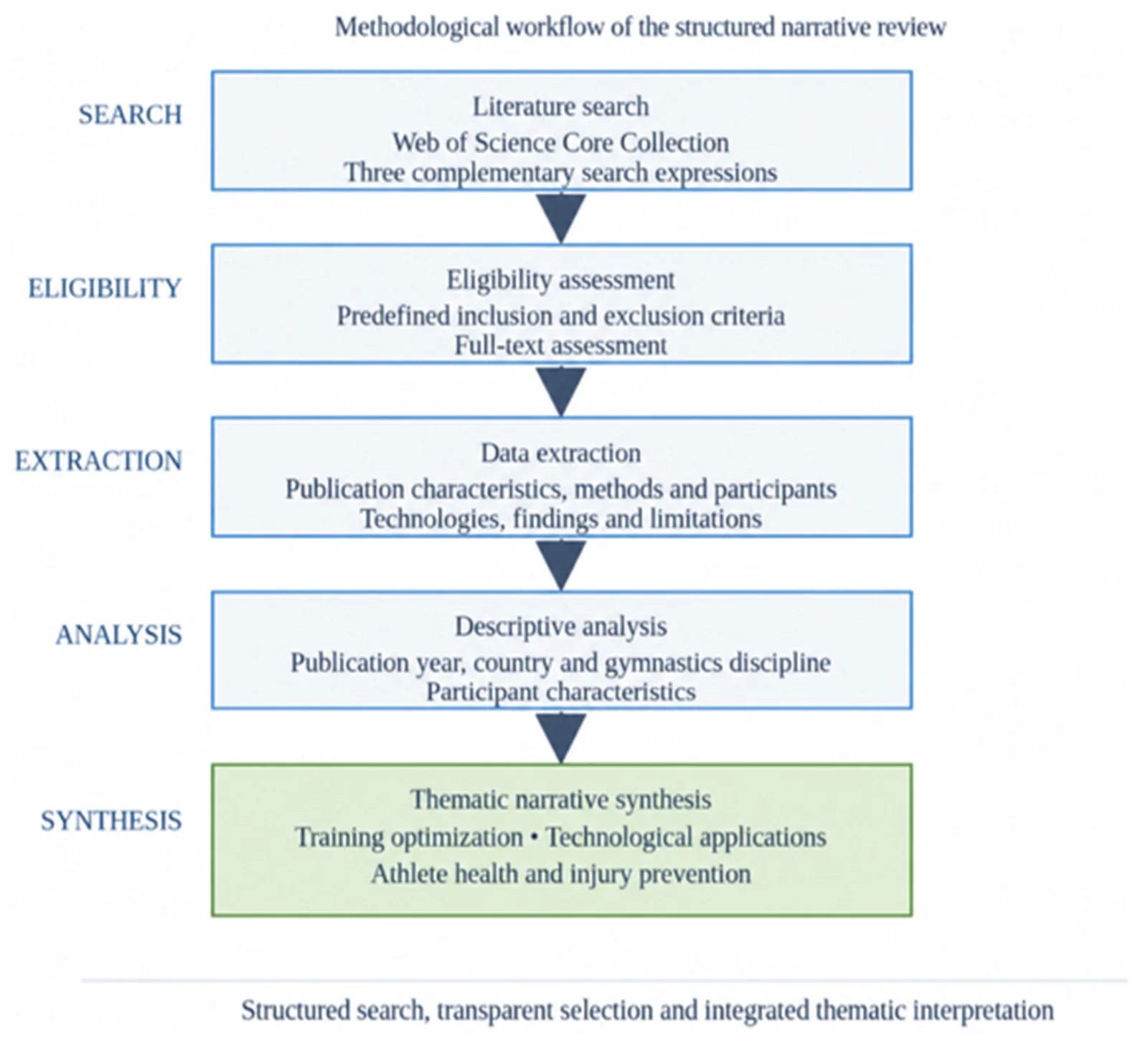
Workflow used for literature identification, eligibility assessment, data extraction, descriptive analysis, and thematic narrative synthesis.

Figure 2 presents the methodological workflow used to conduct the review. It illustrates the progression from literature searching and eligibility assessment to data extraction, descriptive analysis, and thematic narrative synthesis. The figure does not represent a validated training model, clinical framework, or injury-prevention programme.

## 3. Results

### 3.1. Characteristics of the Included Publications

The narrative synthesis included 140 publications published between 2005 and September 2025. Of these, 54 publications were published between 2005 and 2019, representing 38.6% of the included literature, whereas 86 publications were published between 2020 and September 2025, representing 61.4%.

**Figure 3:**
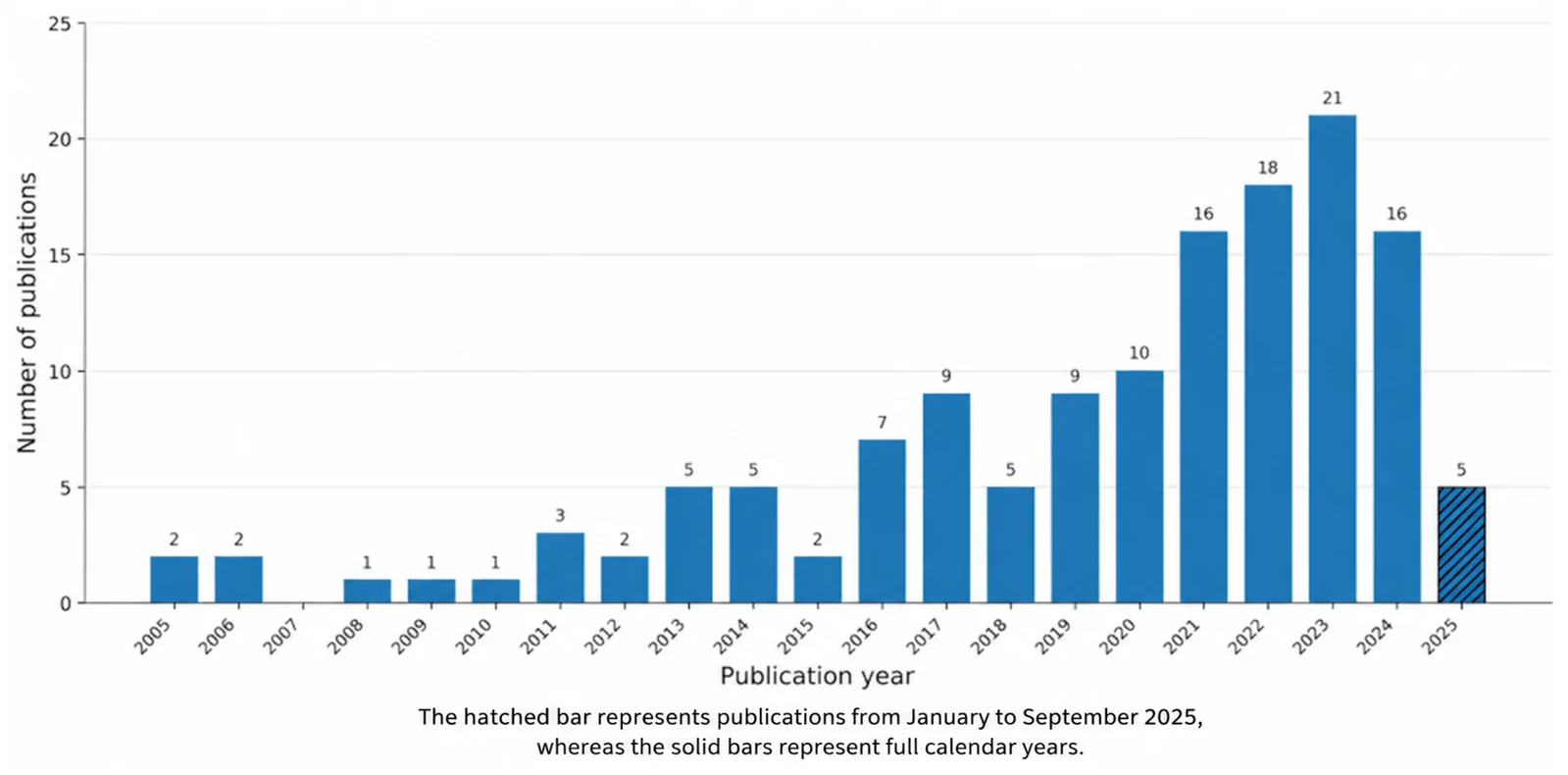
Annual distribution of the publications included in the narrative synthesis. Data for 2025 include publications indexed up to the search cut-off date of 30 September 2025. The hatched bar represents publications from January to September 2025, whereas the solid bars represent full calendar years.

The annual distribution showed an increase in publication activity in recent years. The highest number of publications was recorded in 2023, with 21 publications, representing 15.0% of the included literature. This was followed by 2022, with 18 publications, representing 12.9%. The years 2021 and 2024 each included 16 publications, representing 11.4% per year. Five publications were recorded for 2025, representing 3.6%; this lower number should be interpreted in relation to the search cut-off date of 30 September 2025 (Figure 3).

The individual characteristics of all 140 publications included in the narrative synthesis are provided in Supplementary Table S1.

According to their principal thematic focus, 78 publications addressed gymnastics training optimization, representing 55.7% of the included literature. A further 37 publications focused primarily on technological applications in gymnastics, representing 26.4%, while 25 publications examined athlete health and injury prevention, representing 17.9%.

To further characterize the evidence base, the 140 included publications were mapped according to their principal review domain and methodological design (Table 2). Training optimization showed the most methodologically diverse evidence base, whereas technological applications were predominantly represented by biomechanical/validation studies, and athlete health and injury prevention was primarily represented by cross-sectional/observational and longitudinal studies.

The descriptive characteristics of the included publications are presented in Table 3.

### 3.2. Gymnastics Training Optimization

#### Training Models, Organization and Technical Preparation

The reviewed studies examined training organization, individualized planning, technical preparation and feedback as components of gymnastics training. Structured planning and regular assessment of motor parameters across longer training periods were associated with improvements in motor abilities and competitive performance [72]. Training models also increasingly considered individual morphological, physical, functional, and technical characteristics when determining exercise content and progression.

Individualized approaches were reported in both competitive and adapted gymnastics. A model developed for parallel bars training in gymnasts with Down syndrome used physical and technical parameters to guide preparation and assess execution quality [73]. Similarly, the Artistic Gymnastics Training Guidance Model incorporated morphological, physical, and functional characteristics to assist decisions concerning exercise number, sequence, and difficulty [74].

Feedback and exercise sequencing were also relevant to technical learning. In aerobic gymnastics, self modelling and expert modelling combined with verbal feedback improved the learning and execution of difficult elements [7]. Studies of floor acrobatics further emphasized the organization of preparatory exercises, positive transfer between related skills, and progressive development of technical content [75,76].

Across these studies, training optimization involved structured planning, individualized assessment, progressive technical development, feedback, and the organization of preparatory exercises. The proposed approaches varied across gymnastics disciplines and populations, highlighting the importance of adapting training organization and technical progression to athletes’ individual characteristics and discipline-specific requirements.

### 3.3. Physical, Coordinative, and Physiological Preparation

The evidence concerning physical preparation included randomized trials, controlled interventions, quasi-experimental studies, biomechanical investigations, and cross-sectional analyses. The intervention studies were conducted mainly among young female rhythmic, artistic, and trampoline gymnasts. Two eight-week randomized trials included 44 and 45 young female rhythmic gymnasts, respectively [11,12]. A ten-month controlled intervention included 43 prepubertal female gymnasts [15], while a six-week controlled study included 14 nationally elite junior trampoline gymnasts [19]. A four-week quasi-experimental study included 18 female artistic gymnasts aged 12 to 17 years [77]. This concentration on small samples of young female athletes limits applicability to male gymnasts, adults, recreational participants, and other gymnastics disciplines.

In young rhythmic gymnasts, an eight-week functional core training programme produced larger improvements in selected core-stability tests than regular training, particularly for the bent-knee fall-out and pelvic-tilt assessments [12]. A related randomized study involving 44 rhythmic gymnasts found improvements in countermovement and single-leg countermovement jump outcomes after an integrated core and plyometric programme, with significant differences across the measured jump variables and improved judges’ scores for the stag and split leaps [11]. These findings indicate a positive direction for core stability, explosive strength, and gymnastics-specific jump performance. However, the short intervention periods, single-team recruitment, young age of the participants, and outcome-specific measurement procedures limit generalization. These studies assessed physical and performance outcomes rather than injury incidence.

Longer and more discipline-specific interventions also reported positive changes. A ten-month neuromuscular programme involving 43 prepubertal female gymnasts was associated with improvements in strength, power, speed, and vault performance compared with gymnastics training alone or a non-gymnastics control condition [15]. However, allocation was based on training schedules rather than randomization. In 14 elite junior trampoline gymnasts, a six-week isometric strength programme increased isometric squat peak force by 379 N, corresponding to 22.4%, compared with an increase of 78 N, or 5.0%, in the comparison group. The group-by-time interaction was statistically significant at *p* = 0.032, and the intervention group also improved repeated time-of-flight performance [19]. The small sample and discipline-specific training stimulus limit transfer to other gymnastics populations. These values are reported by the original trampoline study.

Cardiorespiratory preparation was examined in a four-week quasi-experimental study involving 18 young female artistic gymnasts [77]. Adding kettlebell swing training to regular gymnastics practice increased peak oxygen uptake by 8.85 mL/kg/min, with a 95% confidence interval from 2.78 to 14.90 mL/kg/min. The group-by-time interaction was statistically significant at *p* = 0.009. Peak heart rate and post-exercise blood lactate decreased after both training conditions, without clear evidence that these changes were specific to kettlebell training. The small convenience sample, non-random allocation, short intervention, and use of a simulated competition limit the applicability of these findings.

Observational and cross-sectional studies identified differences in stretch-shortening-cycle function, grip strength, postural control, muscle characteristics, and tendon stiffness according to competitive level, apparatus, and training exposure [16,20,69,78]. These studies describe associations and group differences but do not establish that the measured characteristics were caused by a specific training method. Evidence concerning pacing and recovery from CrossFit and related exercise formats [79,80] is indirect. It may inform hypotheses concerning exercise organization, but its applicability to gymnastics-specific training requires direct validation.

Overall, the intervention studies reported changes in a favorable direction for core stability, explosive strength, isometric force, time of flight, cardiorespiratory fitness, and selected gymnastics performance outcomes. The magnitude and consistency of these changes varied according to the intervention, outcome, discipline, and population. Small samples, limited randomization, short intervention periods, heterogeneous outcomes, and the predominance of young female participants restrict direct comparison and generalization across gymnastics settings.

### 3.4. Psychological Preparation and Socio-Emotional Factors

Psychological and socioemotional factors were examined in relation to motor learning, performance, athlete welfare, and continued participation in gymnastics. The reviewed studies addressed self-concept, mental representations, personality, parental involvement, motivational climate, emotional abuse, pain culture, resilience, and coaching behavior.

Self-concept and self-esteem were associated with sporting development, particularly in environments that support both motor competence and confidence [81]. Psychological models also emphasized motivational climates focused on learning and individual progress rather than predominantly on competition and social comparison [82].

The cognitive organization of movement represents another component of motor learning. Research using the Structural Dimensional Analysis of Mental Representation showed that the organization of Basic Action Concepts was related to movement learning and technical development [83]. Mental representations and imagery may therefore complement physical practice during skill acquisition and technical correction.

Personality characteristics have also been investigated in acrobatic gymnastics. Higher performing junior athletes showed greater emotional stability, conscientiousness, and openness to experience [22]. These findings describe associations with performance level but do not establish personality traits as predictors of sporting success.

Several studies focused on the social environment surrounding gymnasts. Emotional abuse can become normalized within youth performance sport when harmful practices are framed as necessary for discipline, commitment, or success [84]. Research in elite women’s artistic gymnastics similarly showed that parents may become accustomed to institutional cultures characterized by intensive training demands and substantial personal sacrifice [23]. Conversely, appropriate parental involvement was associated with achievement motivation, psychological preparation, and continued participation [85].

Pain culture was another concern identified in elite gymnastics. Athletes may learn to consider pain and injury as expected aspects of high-level performance, which can contribute to underreporting or normalization of symptoms [86]. Resilience was associated with adaptation to adversity and recovery following setbacks, with social support playing an important role in this process [87].

Behavioral coaching approaches have used goal setting, observable feedback, performance monitoring, and reinforcement to structure athlete development [88]. Together, the reviewed studies show that psychological preparation in gymnastics extends beyond competition-related mental skills and includes cognitive, emotional, social, and organizational factors that influence both performance and athlete welfare.

### 3.5. Athlete Selection and Performance Evaluation

Athlete selection and performance evaluation involve objective measurements, professional judgement, and consideration of the gymnast’s developmental stage. The reviewed studies examined early selection, motor testing, biological maturation, discipline- specific expertise, judging competence, and factors associated with elite performance.

One study proposed selection criteria for six-year-old children based on height, body mass, body proportions, strength, balance, coordination, and flexibility [89]. These measures may support an initial assessment of characteristics relevant to gymnastics. However, their value for predicting long-term development remains limited because growth, maturation, motivation, learning capacity, coaching, and training opportunities may change over time.

Performance evaluation also depends on professional expertise. In rhythmic gymnastics, judging requires technical knowledge and consistent assessment of artistic and expressive components [90]. Gymnastics coaches evaluated movement execution more accurately than physical education teachers, particularly regarding movement timing and the kinetic features of successful performance [29].

Motor and physical characteristics were associated with participation and performance level. Among children aged 8 to 11 years, gymnastics participation was associated with greater gross motor coordination than cycling and swimming [17]. In rhythmic gymnasts aged 13 to 15 years, flexibility, coordination, and explosive strength differed according to performance level, while biological age influenced the development and expression of these abilities [91].

Elite performance appears to reflect the interaction of multiple characteristics. A multisport study of 296 athletes, which included gymnasts among participants, provided indirect evidence concerning physical, biological, cognitive, psychological, nutritional, social, and training-related factors associated with elite performance [66]. No single profile characterized all elite athletes. Different combinations of strengths were associated with high performance and advantages in some areas appeared to compensate for limitations in others [66].

Together with the gymnastics-specific studies discussed above, this multisport evidence suggests that selection and evaluation in gymnastics should not rely on a single anthropometric measure, motor test, or competitive result. Assessment models need to consider physical and technical abilities together with maturation, learning capacity, psychological characteristics, health, and training conditions.

### 3.6. Physiological and Functional Effects of Gymnastics at Recreational, Corrective, and Competitive Levels

The reviewed studies examined the physiological and functional effects of gymnastics in recreational, corrective, and competitive settings. Reported outcomes included changes in bone health, cardiovascular function, body composition, mobility, coordination, postural control, and selected health indicators. These effects varied according to age, biological maturation, training volume, exercise intensity, and type of gymnastics.

#### 3.6.1. Physiological and Functional Effects of Recreational Gymnastics

Gymnastics exposes the skeleton to multidirectional mechanical forces through jumping, tumbling, landing, and upper limb support. Indirect evidence from comparisons across impact and lower-impact sports indicated greater increases in bone mineral density among adolescents participating in impact sports than in activities with lower mechanical loading, such as swimming [56].

Recreational gymnastics during childhood was associated with favorable bone adaptations in the radius, femur, and lumbar spine, even at relatively low weekly training volumes [57,58]. Participation during periods of rapid growth may also produce skeletal benefits that persist after training has ended [59]. Competitive participation was associated with greater adaptations in some anatomical regions, particularly the forearm and femur, although the magnitude of these changes varied according to training exposure and biological maturation [60,61].

Cardiovascular outcomes were also examined. Indirect evidence from aerobics as a general exercise activity, rather than the recognized competitive discipline of aerobic gymnastics, showed associations with cardiovascular disease risk indicators in a population-based study [62]. Different frequencies and intensities of aerobic exercise were also associated with changes in body composition, body shape, and cardiopulmonary function [35].

The organization of recreational activities may influence functional outcomes. Interactive and game-based activities used during health camps improved spinal flexibility and movement coordination in children and adolescents [63]. Among adult women, a history of moderate-intensity recreational gymnastics was associated with a lower risk of traumatic locomotor disorders than sedentary behavior [38]. These observational findings support an association between long-term participation and the maintenance of strength, balance, coordination, mobility, and physical function.

#### 3.6.2. Functional Effects of Corrective Gymnastics

Corrective gymnastics was examined as part of a combined intervention for children with flatfoot. The programme included corrective exercises and therapeutic swimming, with the involvement of an orthopedic physician, a coach, and the participants’ families. Foot morphology was assessed using the Chizhin index, and favorable changes were reported following the intervention [41].

Because corrective gymnastics and therapeutic swimming were delivered together, the separate contribution of each component could not be determined. The available findings therefore describe the effects of a combined programme rather than corrective gymnastics alone.

#### 3.6.3. Physiological and Functional Effects of Competitive Gymnastics

Competitive gymnastics involves greater training volume, intensity, repetition, and technical difficulty than recreational participation. The reviewed studies identified both physiological adaptations and specific health concerns.

Long-term rhythmic gymnastics training during the prepubertal period was associated with greater bone mass accumulation in girls, despite lower body fat and leptin concentrations. Skeletal development was assessed longitudinally using dual-energy X-ray absorptiometry [64]. These results indicate favorable bone adaptations to repeated mechanical loading during growth, although bone health also depends on energy availability, hormonal function, nutrition, and biological maturation.

Biological markers were examined as potential indicators of training response. Salivary adiponectin increased with training intensity in elite rhythmic gymnasts, but it did not reliably predict reproductive dysfunction or bone mass accumulation [65]. Its value as an independent marker of physiological or health status therefore appears limited.

Pelvic floor health was another concern. Athletes involved in high-impact activities, including trampoline gymnastics, showed a higher prevalence of urinary incontinence than sedentary women. Pelvic floor muscle training increased muscle strength and reduced involuntary urine loss in young nulliparous athletes [92].

The reviewed evidence shows that competitive gymnastics can produce favourable skeletal adaptations while exposing athletes to health concerns related to training load, energy availability, reproductive function, and repeated impact. The reported outcomes varied across disciplines and populations, which limits direct comparison between studies.

### 3.7. Effects of Gymnastics on Physical and Psychological Well-Being Across the Lifespan

The reviewed literature examined the contribution of gymnastics and related physical activity programmes to well-being across childhood, adolescence, adulthood, and older age. The reported effects depended on age, biological maturation, programme structure, accessibility, social context, and adaptation to participants’ functional needs [93].

Assessment tools were developed to support programme planning and service evaluation. One study introduced a computerized multicriteria model using Fuzzy Toolbox software to assess fitness service quality through several indicators [94]. Although developed for fitness services, the model provides a structured approach to evaluating programme organization, accessibility, participant satisfaction, professional competence, and service quality.

Age and biological development influence both physical activity and fitness. Children generally participate in more spontaneous movement, while adolescents engage more frequently in organized activities. However, physical activity commonly declines during adolescence. Fitness during this period reflects interactions among activity level, maturation, growth, and body composition [95]. Gymnastics programmes may support motor competence, balance, coordination, strength, flexibility, body awareness, and confidence, but their content needs to reflect differences in maturation and functional ability.

Participation patterns were also examined among students from the Shaanxi, Gansu, and Ningxia region. A validated model that included gymnastics practitioners identified characteristics of the sporting population and factors associated with participation [36]. This approach may help educational and sports organizations identify participation differences and groups with limited access to organized activities.

Health-oriented programmes have also been studied among adults and older people. The GESTALT compact multimodal programme combined physical activity with social interaction and cognitive stimulation [96]. Its findings support programmes that integrate movement, coordination, mobility, strength, cognitive tasks, and group participation. For older adults, adapted activities may contribute to mobility, postural control, functional independence, and continued social participation.

Digital delivery increased the accessibility of recreational gymnastics during periods of social restriction. Online programmes allowed participants to continue structured exercise at home and increased awareness of regular physical activity [97]. Their effectiveness depended on clear instruction, appropriate exercise selection, supervision, and adaptation of movement difficulty.

Across the lifespan, gymnastics and related programmes were associated with physical, functional, cognitive, and social outcomes. However, differences in programme content, participant characteristics, delivery methods, and outcome measures limit direct comparison between studies.

### 3.8. Contributions of Adapted Gymnastics to Health, Autonomy, and Social Integration

The reviewed studies examined adapted gymnastics and physical education in relation to health, functional capacity, autonomy, and social participation. Programme content varied according to participants’ medical conditions, functional abilities, exercise tolerance, age, and educational needs.

Indirect evidence from individualized physical education programmes indicated improvements in health status, functional ability, exercise tolerance, and participation among university students with health conditions [98]. Adaptations involved changes in exercise content, intensity, volume, execution conditions, instructional methods, and assessment criteria.

Studies involving people with Down syndrome reported that adapted gymnastics may contribute to balance, coordination, strength, mobility, body awareness, and functional independence [39,40]. Structured activities also provided opportunities to follow instructions, learn routines, communicate with others, and assume responsibilities within a group.

Social outcomes were linked to participation in group activities and support from families, teachers, coaches, therapists, mentors, and peers. These settings created opportunities for communication, cooperation, and involvement in shared tasks. However, the reported effects depended on programme accessibility, instructor competence, group climate, individual adaptation, and continuity of participation.

The available studies differed in participant characteristics, intervention duration, programme content, and assessment methods. This heterogeneity limits direct comparison and prevents the identification of a standardized adapted gymnastics programme. Taken together, the gymnastics-specific findings [39,40] and indirect evidence from individualized physical education [98] associate appropriately structured and individualized programmes with physical development, autonomy, communication, and social participation among people with Down syndrome and other functional needs.

### 3.9. The Role of Gymnastics in School and University Physical Education

The reviewed studies examined gymnastics in school and university physical education in relation to motor development, social responsibility, emotional regulation, motor literacy, and teacher preparation. Its educational contribution varied according to curriculum design, task progression, instructional support, and adaptation to learners’ abilities.

At primary school level, a gymnastics and rhythmic activities programme based on the Teaching Personal and Social Responsibility model addressed cooperation, respect, interpersonal conflict, and responsibility through movement, play, and progressive learning tasks. The findings indicated improvements in social behavior without primary reliance on external rewards [33].

Curricular organization was also examined in relation to fundamental motor development. An analysis of primary school curricula and teaching practices identified the need for closer alignment among educational objectives, learning content, and assessment methods. The study emphasized fundamental motor skills that prepare pupils for introductory gymnastics and proposed improvements in curricular organization [99].

At university level, gymnastics contributed to the professional preparation of future physical education teachers. A qualitative study found that students’ bodily and pedagogical experiences during gymnastics classes influenced their understanding of the discipline, professional identity, confidence, and perceived ability to teach gymnastics [100].

Emotional responses also affected learning in university gymnastics courses. Students reported anxiety and perceived lack of competence. Coping responses included reflection and self-regulation, while peer support appeared less frequently [101]. These findings show that emotional regulation, autonomy, and perceived competence form part of gymnastics teacher preparation.

Gymnastics-related exercises were also examined as accessible conditioning activities in university physical education. Romanian research reported that integrating rope exercises into classes provided a low-cost method for developing cardiovascular efficiency and muscular strength among students [102].

A comparison of physical education systems in South Korea, Japan, and the United States showed that gymnastics held a particularly integrated position in the Japanese curriculum. It was connected with physical fitness, fundamental movement patterns, and other sports, supporting its role in motor literacy and broader motor development [103].

The reviewed evidence associates gymnastics in school settings with motor competence and social development, while university programmes contribute to physical conditioning, emotional regulation, professional identity, and teaching confidence. Differences in educational level, curricular structure, teaching methods, and study design limit direct comparison across the available evidence.

### 3.10. Biomechanics, Kinematic Analysis, and Motion Modelling

The reviewed studies examined biomechanical modelling, kinematic analysis, video-based measurement, virtual environments, and motion capture systems in relation to technical execution, motor learning, and performance evaluation in gymnastics. These technologies quantified variables such as joint angles, trajectories, velocities, accelerations, forces, contact times, mechanical work, kinetic energy, muscular activity, and changes in the centre of mass.

A multisegment dynamic model of pommel horse performance combined simulations, three-dimensional movement data, and surface electromyography. The model provided information about muscular coordination and force production during movement execution [104]. Mathematical modelling was also applied to uneven bars dismounts. Biomechanical video analysis identified successive technical phases and relationships between body movements [105].

Computerized biomechanical analysis quantified movement trajectories, speed, acceleration, force, mechanical work, and kinetic energy within gymnastics routines [106]. Kinematic analysis of forward handsprings, backward handsprings, and forward handspring vaults measured joint angles, velocities, and accelerations. Training tasks based on these measurements improved the execution of junior gymnasts and reduced some technical differences between their performances and those of an advanced gymnast [5].

Three-dimensional analysis was used to identify variables associated with successful execution of the Lou Yun vault. Movement was recorded using two digital cameras operating at 50 Hz. A 14-segment body model and the Gubitz method were used to calculate the centre of mass. Short vault-table contact, high vertical velocity, an extended second flight phase, and an appropriate centre of mass height during landing were identified as relevant technical characteristics [107].

Virtual technology was examined as a method for developing visual orientation and landing technique. A virtual environment provided accurate and repeatable feedback about spatial orientation and landing execution [108]. Technology was also incorporated into flexibility training. The Flexibility Trainer combined contract-relax-antagonist-contract proprioceptive neuromuscular facilitation stretching with isokinetic exercise and produced improvements in active and passive hip flexibility [70].

Biomechanical modelling also examined the influence of individual physical characteristics on static strength elements. A two-dimensional, nine-segment model showed that body proportions and centre of gravity location influenced the mechanical difficulty of maintaining the planche position [18].

Motion capture was used to evaluate postural balance in adolescents following a programme based on fundamental gymnastics exercises. Changes in centre of mass position and body stability provided objective information about adaptations in postural control [4].

The reviewed evidence shows that two-dimensional, three-dimensional, and multisegment models can provide objective information about technical phases, force production, muscular coordination, flexibility, balance, and movement efficiency. However, differences in equipment, analytical procedures, movement tasks, and participant characteristics limit comparison across studies and the direct transfer of findings to daily training.

### 3.11. Portable Sensors, Inertial Measurement Units, and Real-Time Monitoring

The reviewed studies examined wearable sensors, motion capture systems, biosensors, auditory feedback, and Internet of Things platforms for movement analysis and technical assessment in gymnastics. These technologies enabled posture recognition, continuous biomechanical monitoring, automated movement comparison, and immediate feedback.

A Kinect-based system captured gymnasts’ movements and analysed body positions in real time. Markov models and similarity calculations compared the gymnast’s execution with a standard movement model. An improved joint reconstruction algorithm used body proportions and geometric principles to estimate joint positions. The system achieved a posture recognition accuracy of 95.7% under the reported validation conditions [2].

Another study developed a mathematical algorithm for detecting gymnasts’ body postures. Motion analysis and mathematical modelling enabled detailed assessment of body positions and provided objective information about technical execution [3].

Sensor feedback was also applied to motor learning. AUFLIP converted movement information into auditory cues during front flip practice. The system was designed to support implicit learning and provide feedback without requiring gymnasts to interrupt the movement or direct their visual attention towards a screen [109].

Advanced motion capture provided detailed spatial and temporal movement data. One system tracked more than 1000 individual points and generated four-dimensional coordinates using standard red, green and blue (RGB) image sensors and passive suits. The method recorded body surface deformation during movement and supported the analysis of body alignment, segment coordination, and movement trajectories [110].

Portable biosensors extended biomechanical monitoring beyond controlled laboratory settings. A validated self-powered sensor recorded biomechanical parameters in real time and demonstrated the potential for continuous technical assessment during movement [9].

Internet of Things technology was used to integrate wearable sensors, wireless communication, cloud-based transmission, and analytical algorithms. One proposed system monitored biomechanical parameters of the lower extremities, particularly the feet and ankles. The recorded data provided information about movement characteristics relevant to performance assessment and the identification of technical potential [25].

The reviewed technologies supported posture analysis, real-time monitoring, motor learning, and objective technical evaluation [2,3,9,25,109,110]. However, the studies differed in sensor type, movement tasks, validation procedures, analytical methods, and testing conditions. These differences limit direct comparison and the generalization of reported accuracy to the full range of complex gymnastics movements.

### 3.12. Artificial Intelligence, Video Analytics, and Deep Learning

The reviewed studies examined artificial intelligence, video analytics, deep learning, and computer vision for performance assessment, movement recognition, technical feedback, and educational applications in gymnastics.

Artificial intelligence was applied to the assessment of physical performance. A neural network model combined with measurements obtained through the My Jump 2 application examined relationships among strength, maximum power, movement speed, anthropometric characteristics, and jumping performance in acrobatic gymnasts [14]. The resulting profiles provided quantitative information relevant to individualized training.

Automated video analysis was used to identify relevant events in gymnastics and other sports recordings. Artificial intelligence systems detected visually distinctive events in unstructured video sequences, including recordings affected by background noise and variations in image quality [111,112]. These methods supported automated event identification and the processing of large video datasets.

Artificial intelligence was also applied to expressive components of performance. Facial emotion recognition networks implemented on mobile devices were proposed for rhythmic gymnastics and figure skating training. These systems analysed athletes’ facial expressions as part of performance monitoring [113].

An artificial intelligence coaching system extracted individual movement trajectories, estimated body postures, detected execution anomalies, and generated visual suggestions for technical correction across video sequences [114]. Artificial intelligence and computer simulation were also used to analyse formation changes in group gymnastics. Big data techniques and information obtained from wireless sensors supported trajectory planning and movement coordination, reducing coordination errors and improving transitions between formations [115,116].

Image processing provided another approach to automated technical assessment. A system based on body contour extraction was developed for movement recognition and analysis, supporting technology-assisted instruction and automated evaluation of gymnastics execution [117]. Digital movement analysis also provided information about body position, trajectories, timing, and coordination for technical feedback [118].

Video feedback was examined during gymnastics skill acquisition. Among beginners, its use was associated with improvements in execution quality, self-assessment, and motivation [119].

More advanced systems applied deep learning to action recognition and classification. Three dimensional convolutional neural networks analysed complex sports movements by processing spatial and temporal information simultaneously [120]. Another approach combined convolutional neural networks for visual feature extraction, Long Short Term Memory recurrent networks for temporal analysis, and a Support Vector Machine for multilabel classification of actions within video sequences [8].

Artificial intelligence was also incorporated into educational approaches. Deep learning and digital technologies were integrated into flipped classroom models in gymnastics and physical education to support personalized feedback, instructional organization, and skill acquisition [32].

Across the reviewed studies, artificial intelligence and video-based technologies were applied to physical performance profiling, movement recognition, event detection, technical correction, expressive assessment, action classification, and education [8,14,32,111,112,113,114,115,116,117,118,119,120]. Differences in datasets, algorithms, validation procedures, recording conditions, and targeted movements limit direct comparison between systems and the generalization of their reported performance.

### 3.13. Technology-Assisted Evaluation and Judging

The reviewed studies examined computerized judging systems, video-based measurement, inertial sensors, machine learning, and artificial intelligence as tools for supporting performance evaluation in gymnastics. These technologies were primarily investigated in relation to scoring accuracy, consistency, objectivity, and technical element recognition.

A Computerised Gymnastics Judging Support System was developed to assist performance evaluation and reduce subjectivity. Stakeholders identified potential benefits related to reduced bias, greater consistency, and improved support for performance assessment. Concerns included system reliability, changes in judges’ professional roles, and dependence on automated decision making [28].

Evidence from diving provides indirect information on video-based performance assessment. Its transferability to gymnastics requires discipline-specific validation. A technological gold standard used measurements of height, angle, and distance, and the resulting rankings were close to those obtained during competition [30]. By analogy, this approach may inform research on technology-assisted gymnastics judging. However, the study did not involve gymnasts or gymnastics judges, and its findings may not be interpreted as direct evidence for gymnastics.

In trampoline gymnastics, an automated system combined inertial sensors with machine learning to detect and classify jumps. The system achieved an accuracy exceeding 96% and demonstrated a high level of consistency in identifying movement events and exercise components [27].

Artificial intelligence was also examined in relation to judging objectivity and professional decision making. The technology showed potential to increase consistency and perceived fairness in performance evaluation. However, its introduction was associated with concerns regarding judges’ professional autonomy, confidence in decision making, and the interpretability of automated outputs [31].

Direct evidence from gymnastics indicates that technology-assisted evaluation can support movement-event recognition, jump classification, and computerized scoring processes [27,28]. Studies from competitive diving and other judged sports provide indirect evidence concerning video-based measurement and artificial intelligence in performance evaluation [30,31]. These findings may inform gymnastics research, but their transferability to official gymnastics competitions requires direct validation. However, differences in technologies, validation procedures, disciplines, measured variables, and testing conditions limit direct comparison between systems and their generalization to official competition settings.

### 3.14. Multimedia Technology, Mobile Learning, and E-Learning in Gymnastics

Multimedia technology, mobile learning, and online educational platforms can support the modernization of gymnastics teaching. These tools combine text, images, audio, video, animation, interactive exercises, and automated feedback. Their contribution is particularly relevant when they improve access to instruction, clarify complex movements, and allow learners to review technical content at their own pace.

Digital technology may complement practical instruction. Gymnastics requires direct movement experience, physical assistance, specific motor feedback, and continuous safety supervision. Consequently, digital resources can support theoretical understanding and technical preparation, but they cannot fully reproduce the interaction between the teacher, the learner, and the physical environment.

Teachers may encounter difficulties when integrating technology into their regular practice. These include limited equipment, inadequate digital competence, insufficient time for preparing resources, technical problems, and uncertainty regarding the pedagogical value of particular applications. Nevertheless, the reviewed studies indicate that digital resources can be integrated effectively into gymnastics and sports education when they respond to clear learning objectives [99,121].

Interactive technologies can help teachers present gymnastics content through demonstrations, sequential images, slow motion recordings, animations, and structured learning tasks. These resources may improve students’ understanding of body positions, technical phases, movement direction, and execution requirements. They can also stimulate teachers to develop instructional strategies adapted to students’ technical level and learning pace.

Online physical education became especially relevant when conventional classes could not take place. Digital platforms allowed teachers to distribute learning materials, demonstrate exercises, maintain communication, and provide some continuity in the educational process. However, these experiences also showed the limitations of remote gymnastics instruction [99,121].

Video communication cannot provide the same level of physical assistance, immediate correction, spatial control, or injury prevention as direct instruction. Camera position, image quality, available space, and internet connection can affect the teacher’s ability to evaluate execution. Students may also perform exercises in environments that lack appropriate surfaces, equipment, or safety measures.

Online gymnastics instruction may therefore focus on activities that learners can perform safely and independently. Suitable content may include mobility exercises, conditioning, basic coordination, posture, balance tasks performed at low height, theoretical analysis, and the observation of technical demonstrations. Complex rotations, inversions, vaulting, apparatus work, and difficult landings require direct supervision and appropriate protective conditions.

Mobile multimedia classrooms provide another method for integrating technology into practical instruction. A system developed according to the Approach to Personalised Teaching model offered immediate feedback, visual materials, and instructional support in real time. Its application in rhythmic gymnastics supported personalised learning and helped improve the organisation of athletes’ technical preparation [122].

The value of a mobile classroom lies in its availability during the practical session. Teachers and coaches can record an execution, replay the relevant phase, compare it with a technical model, and formulate a specific correction. Athletes can then apply the correction during the next attempt. This short feedback cycle may improve the connection between observation, understanding, and motor execution.

Personalised teaching also allows the learning content to reflect individual differences. Students and athletes may differ in previous experience, coordination, flexibility, strength, learning speed, and confidence. Multimedia resources can provide different levels of explanation and technical difficulty. However, the teacher must decide which resource is appropriate and how much information the learner can use effectively.

Digital tools have also been applied to the teaching of choreographic structures. Automated choreography software based on virtual technology can represent movement sequences, spatial formations, directions, timing, and transitions. This visual organisation may help learners understand the relationship between individual movements and the complete composition [123].

Such software may be particularly useful in rhythmic, aerobic, acrobatic, and group gymnastics, where performance depends on the coordination of movement with music, space, apparatus, partners, and formation changes. Coaches can examine several choreographic options before requiring athletes to repeat them physically. This may reduce unnecessary training volume and facilitate communication within the group.

Automated choreography cannot determine every artistic and technical decision. Expressiveness, musical interpretation, group interaction, and the individual characteristics of athletes require professional judgement. Digital modelling may therefore assist the creative process while preserving the role of the coach, choreographer, and athletes.

Continuous animation represents another multimedia method used in gymnastics education. An experimental case study integrated animated movement sequences into teaching and reported improvements in students’ theoretical understanding and practical performance [124]. Animation can divide a difficult movement into successive phases and present details that may be difficult to observe during a normal demonstration.

Learners can pause, repeat, and examine the movement from different perspectives. This may improve their understanding of starting position, direction, timing, body alignment, and final position. However, understanding an animation does not guarantee correct motor execution. Practical learning still depends on physical capacity, kinaesthetic awareness, repetition, feedback, and task progression.

Educational software developed specifically for gymnastics may also increase learners’ interest, satisfaction, and engagement in online instruction [125]. A dedicated platform can organise technical demonstrations, theoretical explanations, movement classifications, safety rules, assessment tasks, and interactive exercises in one learning environment.

The quality of such software depends on the accuracy of its technical content and its compatibility with the curriculum. The material may use correct terminology, clear demonstrations, appropriate progression, and exercises suited to the learner’s age and preparation. Teachers may also verify whether improved satisfaction is accompanied by measurable progress in knowledge and motor performance.

The reviewed literature includes a distance learning platform developed through Flash technology. The platform integrated images, sound, video, and interactive elements. It also used Back Propagation neural networks to adapt or optimise parts of the educational process. Its experimental application among university students suggested that multimedia systems can increase learning efficiency and support the modernization of sports education [126,127].

These findings remain relevant as evidence of the potential value of integrated multimedia learning. However, Flash technology is obsolete and unsupported in current web environments. The results may therefore be interpreted as evidence for the instructional model rather than as support for continued use of that specific technical platform. Current systems may use accessible web standards, responsive interfaces, secure data management, and compatibility with mobile devices.

Back Propagation neural networks may support the analysis of learning patterns and the adaptation of educational content. Their educational value depends on the quality of the input data, the selected outcome indicators, and the transparency of the recommendations. Automated adaptation may assist teachers rather than determine learning decisions independently.

Accessibility represents a central requirement for digital gymnastics education. Educational materials may remain available across different devices, locations, and schedules. This can reduce geographical barriers and support students who cannot attend every direct session. Digital access may be particularly useful for theoretical preparation, revision, teacher education, and communication between training sessions.

Technological accessibility also requires resources that people with different physical, sensory, or cognitive abilities can use. Platforms may provide readable text, captions for video materials, audio alternatives, clear navigation, appropriate colour contrast, adjustable playback speed, and compatibility with assistive technologies. These functions can extend participation and support adapted physical education.

Access remains limited by the cost of equipment and the technical expertise required to operate advanced systems. Artificial intelligence applications, four dimensional motion capture, specialised sensors, and professional analysis software may be unavailable to schools or clubs with restricted resources. Digital inequality can therefore affect who benefits from technological innovation.

Institutions may select technology according to educational value, cost, staff competence, and practical sustainability. A smartphone camera and a well-designed instructional platform may provide greater everyday value than an advanced system that teachers cannot operate consistently. Training for teachers and coaches is therefore essential.

The reviewed evidence suggests potential value in the use of video feedback, multimedia resources, mobile learning, animation, choreography software, educational platforms, and adaptive systems in gymnastics education [99,121,122,123,124,125,126,127]. These technologies may improve access to information, support personalised learning, clarify technical content, and facilitate feedback.

Their effectiveness depends on pedagogical design, technical accessibility, teacher competence, learner safety, and integration with supervised practice. Digital technology contributes most when it supports direct instruction and provides clear information that students and athletes can apply during movement learning.

### 3.15. Multidimensional Syntheses and Perspectives on Technologies in Gymnastics

The reviewed studies provided broader technological, qualitative, and sociocultural perspectives on the use of technology in gymnastics. They examined its contribution to performance assessment, safety, technical development, communication, learning, and the historical development of the sport [24,26,67].

A review of technological applications in gymnastics reported that biomechanical models, wearable sensors, video analysis, artificial intelligence, and digital feedback systems could improve the accuracy of performance evaluation and provide information relevant to athlete safety and technical preparation [26]. These technologies supported the identification of movement deviations, measurement of technical variables, monitoring of training responses, and adaptation of training tasks.

Qualitative research examined how technological feedback interacted with communication between coaches and gymnasts. An ethnographic investigation in rhythmic gymnastics associated the quality of coach-athlete communication and feedback with technical understanding, skill development, and performance [67]. The findings indicated that the contribution of feedback depended on its timing, clarity, interpretation, and integration into the learning process.

Sociocultural research examined the historical and institutional factors underlying differences between women’s and men’s artistic gymnastics. The analysis showed that apparatus selection, competition regulations, social norms, and gender conventions contributed to the development of discipline-specific practices and performance expectations [24]. This perspective indicated that technological systems and performance models operate within existing cultural and regulatory structures.

Across these studies, technology was associated with performance evaluation, safety monitoring, technical preparation, feedback, and learning [24,26,67]. The evidence also connected technological implementation with coach-athlete communication, historical gender conventions, institutional practices, and access to resources. However, the studies differed substantially in purpose, methodology, and level of analysis, limiting direct comparison between their findings.

### 3.16. Injury Prevalence, Patterns, and Prevention

The reviewed studies examined injury prevalence, risk factors, biomechanical loading, functional preparation, rehabilitation, and preventive strategies across several gymnastics disciplines and participation contexts.

Biomechanical analysis identified the lumbar spine as an important injury site. One study examined five men’s artistic gymnastics elements using three-dimensional motion capture, force platforms, and individualised biomechanical modelling. Movements combining unfavourable lumbar positions with high landing forces produced substantial lumbar loading. The round off back handspring and tucked somersaults required particular biomechanical attention [48].

Research involving asymptomatic elite gymnasts provided preliminary normative values for lumbar range of motion. The findings demonstrated the importance of assessing the upper and lower lumbar regions separately because these segments contributed differently to movement and compressive load distribution [128].

Stress fractures represented another concern, especially among physically active girls during growth. Data from the Growing Up Today Study identified gymnastics, running, and basketball as activities associated with stress fracture risk when performed at high volumes. More than eight hours of physical activity per week was associated with approximately twice the risk of stress fracture [129].

Gymnastics-specific core and plyometric interventions in young rhythmic gymnasts reported improvements in balance, movement control, trunk muscle endurance, explosive strength, speed, agility, and jumping performance [10,11,12,130]. These findings concern physical and performance-related outcomes and may not be interpreted as direct evidence of reduced injury incidence.

A cluster-randomized controlled trial involving 23 competitive rhythmic gymnastics clubs evaluated an eight-month gymnastics-specific injury prevention programme. No statistically significant between-group difference was found in the prevalence of overuse injuries in the targeted anatomical locations, with an odds ratio of 0.86, a 95% confidence interval from 0.32 to 2.29, and *p* = 0.77 [42]. The confidence interval was wide and included both a potentially meaningful reduction and an increase in injury prevalence. The result should therefore not be interpreted as evidence of equivalence or as demonstrating the absence of a potentially important intervention effect.

Epidemiological research examined injury characteristics across different gymnastics disciplines. A protocol developed for elite Swedish TeamGym investigated injury patterns, training volume, physical and psychological factors, and normative values for lower limb functional tests. These assessments were also intended to support return to sport decisions [51].

Research on acute injuries in Swedish gymnastics examined injury incidence, anatomical distribution, severity, and financial consequences. Knee and anterior cruciate ligament injuries were particularly important because of their association with prolonged absence, disability, and treatment costs [49]. The included evidence describes the occurrence and consequences of these injuries but does not establish the effectiveness of exercise-based programmes for reducing anterior cruciate ligament injury risk in gymnastics.

Injury incidence and its relationship with training factors were also examined among elite aerobic gymnasts in Spain [131]. A one-year study involving French national artistic gymnasts evaluated injury prevalence and internal and external risk factors. Previous injuries, general health, body weight, psychological condition, stress, anxiety, sadness, and insufficient sleep were associated with injury frequency or recovery capacity [50].

A joint injury prevention protocol in aerobic gymnastics investigated suspension-based rehabilitation and its effects on joint recovery and motor function [53]. Achilles tendon ruptures were examined among university gymnasts, including differences between injuries sustained during training and competition [44]. University gymnastics showed a high injury incidence, although most athletes had a favourable return prognosis. Injury patterns and return rates differed according to sex, apparatus, and discipline- specific demands [132].

Two studies provided information about injury and musculoskeletal pain patterns. A study of calisthenics participants examined injury prevalence, anatomical location, injury mechanisms, and training-related factors associated with the reported injuries [133]. A study involving young female athletes from artistic gymnastics, rhythmic gymnastics, and dance examined the occurrence and anatomical distribution of musculoskeletal pain across different body regions [134]. These publications support the monitoring of injury patterns and musculoskeletal symptoms but do not provide evidence concerning muscle activation or neuromuscular coordination.

The quality and accessibility of online injury prevention information were also assessed. Digital resources provided educational opportunities, but many lacked structured, standardised, and evidence-based content [71].

Studies comparing flexibility methods reported different acute effects on vertical jump performance. Continuous static stretching produced a greater reduction in jumping performance than intermittent static stretching. Dynamic stretching generally produced neutral or favourable effects, while responses to proprioceptive neuromuscular facilitation depended on stretching duration and intensity [46,47].

A broader review associated postural and neuromuscular control with performance in sports requiring precision and fine movement regulation. Neuromuscular and stability exercises improved balance and movement efficiency [13].

Across the reviewed studies, gymnastics injuries were associated with biomechanical loading, training exposure, previous injuries, discipline specific demands, psychological factors, sleep, flexibility practices, and neuromuscular control [13,44,45,46,47,48,49,50,51,53,71,128,129,131,132,133,134,135]. Differences in injury definitions, disciplines, competitive levels, assessment procedures, and study designs limit direct comparison across the available evidence.

### 3.17. Factors Associated with Gymnastics Injuries

The reviewed studies examined biological, biomechanical, technical, training-related, and organisational factors associated with injuries in gymnastics. The main factors included early sport specialisation, biological growth, training load, technical complexity, competition exposure, previous injuries, insufficient recovery, menstrual dysfunction, and inadequate monitoring.

Sport specialisation was investigated among young competitive gymnasts in relation to physical fitness and functional task performance [136]. The study examined differences in physical and functional characteristics according to the degree of specialisation but did not directly evaluate injury incidence or establish sport specialisation as an injury risk factor. Its findings may therefore not be interpreted as evidence of a causal or preventive relationship with gymnastics injuries.

Injury patterns in aerobic gymnastics were reported to resemble those observed in artistic gymnastics [43]. Both disciplines involve repeated jumps, dynamic strength elements, rapid transitions, complex body positions, and high-impact landings. Injury frequency increased with performance level and technical complexity, reflecting longer training duration, more repetitions, difficult skills, and greater competition exposure [43].

Musculoskeletal pain was examined among professional artistic and rhythmic gymnasts. Its prevalence and anatomical distribution were associated with age, duration of sports participation, body mass index, lifestyle, and posture [68]. Longer participation represented greater cumulative exposure to repetitive loading, impact, and previous musculoskeletal problems.

Biological growth and training load were investigated among tumbling and elite gymnasts during competitive seasons [54,137]. Accelerated growth was associated with changes in body proportions, coordination, strength, flexibility, and centre of mass position. Rapid increases in volume, intensity, technical difficulty, or competition exposure were associated with fatigue and reduced recovery capacity [54,137].

Foot and ankle injuries were frequently reported in jumping sports. Gymnasts experienced a considerable number of overuse injuries, while acute accidents occurred more frequently during competitions than during training [138]. Repeated take-offs, rebounds, rotations, balance positions, and landings exposed the foot and ankle to substantial mechanical demands.

Bone stress injuries represented another concern, especially among adolescent athletes. A broad review identified high mechanical loading, rapid growth, inadequate recovery, low energy availability, menstrual dysfunction, and previous bone stress injuries as relevant risk factors [139]. These injuries developed when repeated loading exceeded the capacity of bone tissue to remodel and recover.

The wrist was identified as a specific injury site in artistic gymnastics. Repeated hand support during handstands, vaulting, tumbling, and apparatus work exposed the wrist to compression, torsion, hyperextension, and impact [140]. Young gymnasts were particularly vulnerable because the distal radial growth plate remained open. Repetitive stress during this developmental period was associated with pain and structural changes [140].

Overuse injuries were also common in rhythmic gymnastics. A study using the Triad-Specific Self-Report Questionnaire identified a higher risk among younger gymnasts and those without menstruation [55]. The knee, lumbar spine, and hip were reported as important anatomical regions. The demands of extreme flexibility, repeated jumps, pivots, balances, and high training volume contributed to injury exposure [55].

Long-term injury consequences were examined among former British Olympians. Medically treated injuries occurred most frequently in the knee, lumbar spine, and shoulder, while gymnastics was among the sports with the highest injury rates [141]. Approximately 20% of the athletes included in the study retired prematurely because of injury. Previous injuries were also associated with chronic pain, osteoarthritis, and joint replacement later in life [141].

Coaches’ knowledge and monitoring practices were examined among professionals working with gymnasts aged 9 to 16 years [52]. The study identified differences between general awareness of growth-related risks and the practical monitoring of maturation and training load. Trampoline coaches commonly recorded external load through training logs and the number of contacts with the trampoline bed. Formal monitoring was less frequent in artistic gymnastics because of the variety of apparatuses and technical elements [52]. Few coaches used structured procedures to estimate peak height velocity or identify periods of accelerated maturation.

Across the reviewed studies, gymnastics injuries were associated with biological growth, excessive or rapidly increased training load, insufficient recovery, technical complexity, competition exposure, previous injuries, menstrual dysfunction, and limited monitoring [43,52,54,55,68,137,138,139,140,141]. Anatomical patterns differed according to discipline-specific demands. Hand-support elements increased wrist exposure, while jumps, rotations, and landings increased loading of the lower limbs and lumbar spine. Differences in study populations, injury definitions, disciplines, and assessment procedures limit direct comparison across studies.

## 4. Discussion

To facilitate interpretation of the findings, the robustness of the evidence is considered qualitatively throughout this Discussion, taking into account the consistency of findings across studies, methodological design, sample size, and the extent of independent replication. Given the structured narrative design of this review and the methodological heterogeneity of the included literature, no formal certainty-of-evidence grading system was applied. Accordingly, findings supported across multiple studies are distinguished, where possible, from those based primarily on isolated studies, small samples, or exploratory research, which are interpreted more cautiously as limited or preliminary evidence. In the absence of a formal methodological quality or risk-of-bias assessment, the practical considerations derived from this synthesis should therefore be interpreted as evidence-informed proposals rather than as evidence-based clinical guidelines or standardized recommendations.

This review examined three connected dimensions of contemporary gymnastics: modern training methodologies, technological systems used to improve training and evaluation, and current approaches to injury prevention. The reviewed evidence suggests that performance optimisation may involve interactions between scientific planning, physical and technical preparation, psychological support, technological feedback, and health monitoring. Rather than functioning as separate interventions, these elements may be considered together within a training process adapted to the gymnast’s age, discipline, competitive level, and individual characteristics.

The findings related to the first objective show that modern gymnastics training requires systematic planning and continuous evaluation. Training optimisation may be supported by the selection of suitable exercises, the organisation of training content, and the adjustment of workload according to individual responses. Scientific control allows coaches to compare planned and completed work, identify changes in performance, and modify training before fatigue or technical errors become persistent [72].

The Artistic Gymnastics Training Guidance Model represents an example of a structured approach that connects performance objectives with training content and evaluation [74]. Such models can increase the consistency of long-term preparation. Their value lies in supporting coaching decisions through clearly defined indicators. However, a model cannot be applied identically to every gymnast. Its recommendations must reflect the athlete’s training history, biological development, apparatus requirements, and current functional capacity.

The development of strength, power, coordination, and technical control remains central to gymnastics performance [15,16,142]. These qualities interact during almost every technical element. Strength supports force production and body control. Coordination determines the temporal and spatial organisation of movement. Flexibility allows the gymnast to reach positions required by the technical code and the aesthetic characteristics of the discipline [70]. An isolated improvement in one capacity does not automatically produce better performance. Excessive flexibility without active control may reduce joint stability, while strength developed without technical integration may have limited transfer to competitive routines.

Taken together, these findings are consistent with an organizing perspective in which physical and technical preparation are considered in combination rather than as isolated components. Physical exercises may reproduce relevant movement patterns, force directions, contraction types, and coordination demands. At the same time, general conditioning remains necessary because it creates the functional foundation for technical learning. Coaches may combine general preparation with apparatus-specific exercises and progressively increase complexity.

Technical learning also depends on the quality of feedback. Research on aerobic gymnastics indicates that structured feedback can improve the acquisition of technical elements [7]. Feedback becomes useful when it identifies the most relevant error and provides an applicable correction. Too much information after every attempt may reduce the gymnast’s ability to process the correction and recognise errors independently.

Positive transfer between related exercises can support technical development [75,76]. Preparatory drills may reproduce essential characteristics of the target skill without exposing the athlete to its complete difficulty. This allows gymnasts to develop body position, timing, rotation, take-off, support, or landing control progressively. The transfer remains effective only when the preparatory exercise reflects the decisive components of the final element.

The available evidence also suggests that modern gymnastics methodologies have applications beyond elite sport. Adapted gymnastics can support health, autonomy, motor competence, and social participation. Objective assessment helps teachers and specialists select tasks that correspond to individual functional possibilities, including those of people with Down syndrome or other specific needs [73]. The aim in this context is not the replication of competitive standards. Training may support participation, independence, confidence, and functional improvement.

Gymnastics also contributes to school and university physical education. It develops body awareness, posture, balance, coordination, strength, mobility, and movement control [101,102,103]. These benefits depend on suitable progression and safe instruction. Teachers may adapt tasks to students’ age, previous experience, and physical capacity. Basic gymnastics can provide valuable motor education without requiring advanced acrobatic elements.

The second objective concerned the technological systems used in gymnastics. The findings show rapid development in biomechanical analysis, wearable sensors, video processing, artificial intelligence, automated movement classification, and digital learning. These technologies increase the amount and precision of information available to coaches, athletes, teachers, and judges [8,14,32,111,112,113,114,115,116,117,118,119,120].

Biomechanical and kinematic analysis can quantify body angles, angular velocity, displacement, rotation, flight time, contact time, and landing characteristics. These measurements can reveal differences that remain difficult to identify through direct observation. They also allow specialists to compare attempts and determine whether a technical correction produces the intended mechanical effect.

Wearable sensors and inertial measurement units offer an additional advantage because specialists can use them in practical training environments. They can record acceleration, orientation, movement timing, and impact-related variables without requiring a complete motion capture laboratory. However, measurement accuracy depends on sensor placement, calibration, sampling frequency, data-processing procedures, and the movement being analysed.

Artificial intelligence extends these possibilities through automated event detection, movement classification, performance prediction, and personalised feedback [8,14,32,111,112,113,114,115,116,117,118,119,120]. Video-based systems can process a large number of executions and identify movement patterns with greater speed than manual analysis. Machine-learning methods may also assist coaches in detecting technical inconsistencies or changes associated with fatigue.

The practical contribution of artificial intelligence depends on the quality and representativeness of its training data. A model developed using a limited group of elite gymnasts may perform poorly when applied to children, beginners, adapted gymnastics participants, or athletes from another discipline. High classification accuracy in controlled conditions does not guarantee equivalent performance during training or competition. Researchers may validate systems across different athletes, technical levels, apparatuses, recording conditions, and movement variations.

Overall, the evidence for artificial intelligence applications in gymnastics may currently be considered preliminary. Although the reviewed studies demonstrate promising applications in movement classification, performance assessment, and automated analysis, the evidence remains heterogeneous and relatively limited in terms of independent replication across gymnastics disciplines, athlete populations, and real-world training or competition settings.

Technology may provide information that coaches can interpret and apply. A system that generates numerous indicators without identifying their practical meaning may complicate decision-making. Coaches may select a small number of variables connected to the current training objective. For example, landing analysis may focus on impact, trunk control, and lower-limb alignment. Rotation analysis may focus on take-off velocity, body configuration, and time available for opening before landing.

The reviewed evidence indicates potential applications of technology in performance evaluation and judging. Direct gymnastics evidence concerning computerized judging-support systems and automated trampoline jump classification is provided by Leukel, C. et al. [29] and Luedeker, B. et al. [30]. An automated system for trampoline gymnastics achieved a classification accuracy exceeding 96% under the reported testing conditions [27]. Indirect evidence from competitive diving showed that a technological gold standard based on movement height, angle, and distance produced rankings close to those obtained during competition [30]. Together with the direct gymnastics evidence [27,28], these findings illustrate the potential of automated assessment, although the transferability of the diving findings to gymnastics requires discipline-specific validation. These results demonstrate the potential of automated assessment, but they do not support the complete replacement of judges.

Gymnastics evaluation includes technical, artistic, and contextual components that current systems may not capture fully. Judges also need to interpret unusual situations, technical failures, and interactions between several execution criteria. Automated systems may provide additional information for performance evaluation, but their validity and practical usefulness must be established separately within each gymnastics discipline [27,28,30,31].

Indirect evidence from other judged-sport contexts suggests that the introduction of technological judging may affect professional autonomy and trust [31]. Judges may resist a system when they cannot understand how it calculates a result. Interpretability is therefore essential. The system may indicate which variables it measured, how these variables affected the evaluation, and when the result may be unreliable. Sports organisations must also establish procedures for technical failures, appeals, conflicting results, and responsibility for the final decision.

Digital technology can modernise gymnastics education. Multimedia classrooms, mobile applications, animations, automated choreography software, and online platforms can clarify technical content and provide repeated access to demonstrations [99,121,122,123,124,125,126,127]. Learners can examine movements in slow motion, compare executions, and review technical phases at their own pace.

Mobile learning can shorten the interval between execution and correction. A teacher can record an attempt, show the relevant phase, formulate a correction, and allow the learner to apply it immediately. This process may improve understanding and support personalised instruction [122]. Animation and specialised software can also represent body positions, movement sequences, formations, and transitions that are difficult to observe during normal-speed demonstrations [123,124,125].

Digital instruction cannot fully replace supervised practical learning. Gymnastics requires physical assistance, direct feedback, equipment control, and continuous safety management. Online instruction may be more appropriate for theoretical preparation, movement analysis, conditioning, mobility, and low-risk coordination tasks [99,121]. Complex rotations, inversions, apparatus work, and high-impact landings require suitable facilities and direct specialist supervision.

Some reviewed studies used platforms that are now obsolete, including Flash-based systems [126,127]. Their findings remain relevant for understanding multimedia and adaptive learning, but they do not validate the continued use of those technical platforms. Current educational systems may use accessible and secure technologies that function across computers and mobile devices.

The broader evidence indicates that technological integration also has ethical and social implications [24,26,67]. Wearable devices, video systems, and artificial intelligence platforms may collect detailed information about movement, health, behaviour, and performance. Athletes must understand what data are collected and how they will be used. Data protection requires particular attention when the gymnasts are minors.

Technology may also reproduce existing assumptions. Algorithms trained using historical routines, judging decisions, or discipline-specific standards can preserve previous biases. This issue is relevant when models influence selection, evaluation, or technical recommendations. Researchers and sports organisations may examine whether the system reflects valid performance criteria and whether it functions equitably across groups.

The third objective addressed injury prevention. The reviewed findings indicate that gymnastics injuries are associated with multiple interacting factors, including mechanical loading, technical complexity, biological growth, previous injury, psychological condition, energy availability, and insufficient recovery [43,44,45,46,47,48,49,50,51,52,53,54,55,68,128,129,131,132,133,134,135,137,138,139,140,141]. The multifactorial nature of gymnastics injuries therefore suggests the potential value of a coordinated approach rather than reliance on a single isolated programme.

Biomechanical analysis can identify movement situations that increase tissue loading. Research on lumbar loading showed that gymnastics elements combining unfavourable spinal positions with high forces may impose substantial mechanical demands [48]. Segment-specific lumbar assessment can provide more useful information than total range of motion because a large movement amplitude may conceal excessive loading of particular segments [128].

Landings remain relevant because gymnastics injury surveillance identifies knee and anterior cruciate ligament injuries as clinically important conditions associated with substantial absence from training and competition [49]. However, the reviewed corpus does not provide direct evidence that landing exercises or neuromuscular programmes reduce anterior cruciate ligament injury incidence in gymnasts. Such interventions may therefore be regarded as an area for future gymnastics-specific research rather than as an established preventive strategy.

Training load must remain compatible with the gymnast’s capacity to recover. Participation in more than eight hours of weekly physical activity was associated with approximately twice the risk of stress fracture in the population examined by one study [129]. This value may not function as a universal threshold for gymnasts. It demonstrates that exposure becomes clinically relevant when training volume interacts with growth, nutrition, maturation, and recovery.

Rapid increases in training volume, intensity, or technical difficulty may be associated with increased injury risk [54,137]. Camps, precompetition periods, new routines, and return after illness or injury represent common situations in which load may rise faster than the athlete’s capacity. Coaches may monitor changes in workload and the gymnast’s response rather than rely only on total weekly hours.

Biological growth requires specific attention. Changes in limb length, body mass distribution, centre of mass, strength, and coordination may temporarily affect technical consistency [52,54,137]. During accelerated growth, coaches may need to reduce repetition density, reinforce basic technique, and adjust physical preparation. Temporary difficulty with previously stable skills may not automatically be interpreted as reduced motivation.

Sport specialisation also requires careful interpretation. Reference [136] examined specialisation in relation to fitness and functional task performance but did not directly assess injury risk. Therefore, the study does not establish that sport specialisation causes gymnastics injuries. Decisions concerning early participation, year-round training, technical progression, motor diversification, nutrition, and recovery may be individualized and informed by the gymnast’s age, maturation, health, and training demands.

Injury patterns differ among disciplines and anatomical regions. Repeated upper-limb support increases wrist stress, especially while the distal radial growth plate remains open [140]. Jumps and landings expose the foot, ankle, knee, hip, and lumbar spine to repeated forces [49,55,138]. Rhythmic gymnastics presents specific risks connected with extreme mobility, repeated jumps, high training volume, and menstrual dysfunction [55]. Bone stress injuries become more likely when high loading interacts with low energy availability, menstrual disturbances, growth, and inadequate recovery [139].

Compared with several emerging technological applications, the injury-related literature provides more consistent evidence concerning the multifactorial nature of injury risk in gymnastics. However, evidence regarding the effectiveness of specific preventive interventions remains limited and heterogeneous across disciplines, age groups, and competitive levels. In a cluster-randomized controlled trial involving competitive rhythmic gymnasts, the estimated effect of an eight-month gymnastics-specific injury prevention programme was not statistically significant, with an odds ratio of 0.86 and a 95% confidence interval from 0.32 to 2.29 [42]. The wide confidence interval indicates considerable uncertainty and does not establish equivalence between the groups. It also does not exclude a potentially meaningful reduction or increase in overuse injury prevalence. Further adequately powered trials are needed before conclusions can be drawn regarding programme effectiveness.

The long-term consequences strengthen the need for early intervention. Research involving former Olympic athletes identified chronic pain, osteoarthritis, joint replacement, and premature retirement following previous injuries. Approximately 20% of the athletes in that study retired prematurely because of injury [141]. Performance planning may therefore consider long-term musculoskeletal health, not only immediate competition participation.

Stretching practices also require suitable placement within the training session. Continuous static stretching may temporarily reduce explosive performance more than intermittent static stretching, while dynamic stretching generally produces neutral or favourable acute effects [46,47]. Gymnasts still require high mobility, but longer static stretching may be more appropriate after explosive work or during separate flexibility sessions.

The findings across all three objectives converge on one central principle. Effective gymnastics preparation requires individualised decisions supported by reliable information. Scientific planning provides the structure. Technology may improve measurement and feedback, while injury surveillance may support the gymnast’s capacity to train and perform. None of these components can replace the knowledge of coaches, teachers, judges, medical specialists, or the athlete’s own experience.

The reviewed evidence identifies converging themes suggesting the potential value of combining technological data, professional judgement, and athlete feedback within the same decision-making process [24,26,67]. This organizing perspective may help readers consider how technological information, professional judgement, athlete feedback, training demands, and health-related observations interact. It does not prescribe a standardized decision-making model or establish the effectiveness of a specific integrated programme.

The main contribution of this review is the organization of selected evidence from training methodology, technological applications, and athlete health and injury prevention within a common interpretative perspective. The reviewed studies generally examined these areas separately, and no included study tested them as one integrated programme. The proposed perspective therefore identifies conceptual links across the three domains but may not be interpreted as an established or validated model. Its practical value and effectiveness require direct evaluation in future gymnastics-specific research.

These results support the three initial objectives. Modern training methodologies may support performance optimisation when they integrate physical, technical, psychological, and educational components [7,15,16,70,72,73,74,75,76,101,102,103,142]. Technological systems may support the efficiency and accuracy of training, learning, and evaluation when specialists use valid and interpretable measurements [8,14,24,26,27,28,32,67,99,111,112,113,114,115,116,117,118,119,120,121,122,123,124,125,126,127]. Evidence transferred from other sports may be treated as indirect until it is validated in gymnastics. The reviewed evidence suggests that injury-prevention planning may consider biomechanics, workload, maturation, recovery, health, and discipline-specific exposure [13,43,44,45,46,47,48,49,50,51,52,53,54,55,68,71,128,129,131,132,133,134,135,136,137,138,139,140,141].

The robustness of this evidence varies across the three domains: some training and injury-related findings are supported across multiple studies, whereas several emerging technological applications, particularly those involving artificial intelligence and automated assessment, remain preliminary and require further independent validation.

Together, these findings provide an evidence-informed basis for safer and more individualised gymnastics practice within the scope of the reviewed literature.

### 4.1. Practice Considerations Within the Reviewed Evidence Corpus

The following considerations are limited to the evidence identified through the focused search strategy used in this review. They represent author-derived interpretations of the included literature and should not be understood as clinical guidelines, consensus recommendations, or practices whose effectiveness has been established across all gymnastics disciplines, age groups, and participation levels. Their application requires professional judgement and adaptation to the gymnast’s age, health status, discipline, training level, and available resources.

Coaches may consider establishing clear objectives for each training stage and selecting a limited number of relevant performance indicators. These indicators can include technical execution, landing control, movement amplitude, training volume, pain, fatigue, sleep quality, and perceived recovery. Regular assessment may help coaches identify negative changes and adjust the programme before they affect performance or health.

The reviewed evidence suggests that physical preparation may combine strength, power, coordination, mobility, balance, trunk stability, and neuromuscular control. Exercises may be selected to reflect the mechanical and coordinative demands of the target gymnastics elements. General conditioning may provide the foundation for apparatus-specific preparation, while a progressive increase in technical difficulty can be considered once the gymnast demonstrates adequate control during simpler tasks.

The reviewed evidence identifies potential value in the use of preparatory exercises designed to reproduce the decisive components of the final skill. These can include take-off mechanics, body configuration, rotation timing, upper-limb support, spatial orientation, or landing position. Progressive learning may reduce unnecessary exposure to difficult elements and facilitate the transfer between physical preparation and technical execution.

Technological feedback can be particularly useful when it addresses a specific training question. Video analysis, wearable sensors, motion capture, and artificial intelligence systems may help specialists measure body angles, flight time, contact time, rotation, impact, and movement consistency. Limiting feedback to one or two corrections linked to the current objective may facilitate a clearer response from the gymnast, although the optimal amount of feedback may vary according to the athlete and training context.

Immediate video feedback may support technical learning when the gymnast actively participates in the analysis. The athlete may be encouraged to identify the difference between the intended and completed movement, describe the perceived execution, and formulate the next correction with the coach. This process may contribute to the development of self-assessment and reduce dependence on external feedback.

Technological measurements benefit from standardized procedures. Where applicable, specialists may consider maintaining consistent camera positions, sensor placement, calibration methods, sampling conditions, and data processing. Measurements collected under different conditions may not permit valid comparisons. The validity and reliability of the selected device for the movement and population being assessed may also be considered when interpreting the resulting data.

Artificial intelligence systems may be used to support professional decisions, where appropriate, but the reviewed evidence does not support their independent use for decision making. Their outputs may be interpreted in relation to the variables measured, input-data quality, model limitations, and the specific training or competition context.

Digital tools may also support gymnastics education. Teachers may use slow-motion video, mobile applications, animation, and online demonstrations, where appropriate to explain movement phases. However, the available evidence does not remove the need for direct supervision, equipment control, progressive assistance, and immediate safety management during practical lessons. Unsupervised digital learning may therefore be more appropriate for theoretical content and low-risk activities.

Athlete health and training safety may be supported through routine monitoring. Coaches may consider recording training duration, the number of high-impact landings, difficult repetitions, complete routines, competition exposure, pain, fatigue, and recovery. Such information can be interpreted in relation to changes from the gymnast’s usual pattern. A rapid increase in volume, intensity, or technical difficulty may indicate a need to consider temporary programme adjustment.

Training-load management may also account for biological maturation. During periods of accelerated growth, gymnasts may experience changes in coordination, flexibility, strength, body proportions, and technical consistency. Periodic monitoring of growth may assist coaches in deciding whether adjustments to repetition density, physical preparation, and skill progression are appropriate when substantial changes occur.

Specialist medical evaluation may be appropriate when the gymnast reports persistent localised pain, swelling, loss of function, recurrent instability, or declining technical control. Persistent pain may warrant further assessment rather than being treated as an expected consequence of training. Early assessment may be particularly relevant for the wrist, lumbar spine, knee, ankle, and areas exposed to bone stress.

Landing training can be considered an important component of physical and technical preparation. A progressive approach can begin with trunk control, hip stability, knee alignment, ankle stability, and balanced force absorption during simple bilateral landings. Progression can then include unilateral tasks, rotations, increased height, apparatus-specific situations, and controlled fatigue.

Mobility training may aim to develop both range of motion and active control. Extreme flexibility without sufficient strength may increase joint stress. Dynamic mobility can be considered before explosive and acrobatic tasks. Longer static stretching can be included after training or in separate flexibility sessions where appropriate and when immediate power production is not a priority.

Recovery may be considered alongside training load. Coaches can consider sleep, nutrition, hydration, planned rest, psychological condition, and menstrual health. Menstrual irregularities, persistent fatigue, recurrent pain, and reduced performance may be associated with inadequate energy availability or insufficient recovery and may warrant specialist assessment.

Return-to-gymnastics decisions may be informed by a combination of functional and technical criteria, including mobility, strength, balance, landing control, apparatus-specific capacity, and confidence; absence of pain alone may not establish readiness for full training or competition.

Judges and sports organisations may consider automated systems as tools for supporting the assessment of measurable aspects of execution, such as height, angle, distance, timing, and body position. Where such systems are implemented, transparent procedures may be appropriate for technical failures, appeals, conflicting assessments, and responsibility for the final score.

Automated systems may complement professional judgement, but the available evidence does not support replacing professional assessment with automated outputs.

Sports organisations may also consider appropriate safeguards for athletes’ personal data, including transparent procedures for data collection, access, storage, and use, particularly when minors are involved.

The reviewed literature identifies potential value in cooperation among gymnasts, coaches, teachers, judges, sports scientists, medical personnel, psychologists, and families. Shared monitoring may help connect technical performance with workload, recovery, growth, and health. Within the limitations of the available evidence, such an approach may support individualised training, informed decisions, safer participation, and long-term performance development.

### 4.2. Limitations

This review has several limitations that may be considered when interpreting its findings.

First, the literature search was conducted exclusively in the Web of Science Core Collection and was restricted to the Sport Sciences category. Some retrieved records also had additional Web of Science category assignments, but no separate searches were conducted in medical, health-related, educational, biomechanical, engineering, or technological categories. Consequently, relevant publications indexed exclusively outside the Sport Sciences category may have been missed.

The findings may therefore not be interpreted as representing the complete multidisciplinary literature on gymnastics.

Second, the search strategy used three focused exact-phrase queries corresponding to the main thematic areas of the review. These queries increased search specificity but may have omitted publications that used alternative terminology, synonyms, broader concepts, or discipline-specific descriptions. The review may therefore be interpreted as a structured narrative synthesis of a focused evidence corpus rather than an exhaustive systematic review of all available research.

Third, only English-language publications were considered, which may have introduced language bias by excluding relevant studies published in other languages. Although full texts were successfully retrieved for all 153 publications selected for eligibility assessment, the restriction to English-language publications may have limited the international coverage of the evidence corpus. This limitation may be particularly relevant in gymnastics research, where important contributions can appear in national or regional journals.

Fourth, the review included publications with considerable methodological heterogeneity. The included literature comprised experimental, observational, descriptive, technological, pedagogical, and review based publications. Differences in participant characteristics, gymnastics disciplines, participation contexts, educational or health-related applications, training levels, intervention duration, outcome measures, and analytical procedures prevented direct quantitative comparison and meta-analysis. The findings were therefore synthesized narratively and may be interpreted as an overview of research directions rather than as pooled evidence of intervention effectiveness.

Fifth, no formal study-level methodological quality or risk-of-bias assessment was incorporated into the predefined methodology of this structured narrative review. Although the included publications were heterogeneous in terms of study design, population, outcomes, and research purpose, design-specific appraisal tools could have been applied to evaluate methodological robustness. Consequently, studies with different levels of methodological strength contributed to the narrative synthesis without formal quality-based weighting. The findings may therefore be interpreted as a structured synthesis of the available evidence rather than as definitive evidence of intervention effectiveness. In particular, practical implications related to injury prevention, workload management, recovery, menstrual health, technological applications, and athlete monitoring may be considered in light of this limitation.

Sixth, the assignment of each publication to one principal thematic area required interpretative judgment. Some publications addressed more than one topic, such as technology supported training combined with injury prevention or performance monitoring. Each publication was assigned to its predominant thematic category for the descriptive analysis to prevent double counting. However, this procedure may have simplified the multidimensional contribution of certain studies. Publications could still contribute to several components of the narrative synthesis.

Seventh, the literature search covered the period from 2005 to 30 September 2025. Consequently, the number of publications recorded for 2025 represents only part of the year and may not be compared directly with complete annual totals. The rapid development of wearable sensors, artificial intelligence, virtual reality, markerless motion analysis, and athlete monitoring systems may also reduce the long-term currency of some technological findings. In addition, restricting the search to 2005–2025 may have excluded relevant foundational studies published before 2005, particularly in established areas such as gymnastics training, injury epidemiology, maturation, bone health, and biomechanics.

Eighth, the descriptive analysis focused on publication year, publication period, and principal thematic area. Additional characteristics, including country, gymnastics discipline, participation context or application, participants, study design, main findings, and reported limitations, were extracted at the publication level and presented in Supplementary Table S1. However, incomplete reporting in some original publications may have restricted the precision of this classification.

Future research may use searches across multiple bibliographic databases and broader combinations of controlled vocabulary and free text terms. Updated reviews may also include discipline specific terminology and, where feasible, publications written in languages other than English. Greater methodological consistency is needed in the reporting of participant characteristics, training exposure, competitive level, intervention protocols, injuries, health outcomes, and technological validation procedures. Future systematic reviews may incorporate appropriate design-specific methodological quality or risk-of-bias assessment tools and, where sufficiently homogeneous data are available, conduct meta-analyses. Longitudinal and multicenter studies are also needed to determine whether emerging training and monitoring technologies produce sustained improvements in performance, health, and injury prevention across different gymnastics disciplines and competitive levels.

### 4.3. Future Research Directions

Future research may use longitudinal and prospective designs to determine how modern training methods influence technical development, performance, health, and athlete retention over several training seasons. These studies may include repeated assessments of physical capacity, technical execution, workload, maturation, recovery, psychological state, and injury occurrence.

Researchers may recruit larger and more diverse samples. Multicentre studies could include gymnasts from different countries, competitive levels, age groups, and disciplines. This approach would improve statistical power and show whether the findings apply beyond a single club, team, or national programme.

Studies involving young gymnasts may assess biological maturation rather than rely only on chronological age. Researchers may examine how growth rate and maturation interact with training load, technical progression, coordination, pain, and injury risk. This information could support age-appropriate training models during periods of accelerated growth.

Experimental studies may compare different combinations of strength, power, mobility, balance, trunk stability, neuromuscular training, and apparatus-specific preparation. Researchers may report the content, intensity, frequency, duration, progression, adherence, and adverse events associated with each intervention. Standardised reporting would support replication and comparison.

Future technological research may establish common protocols for camera placement, sensor attachment, calibration, sampling frequency, data processing, and performance indicators. Studies may report measurement validity, reliability, sensitivity to change, and practical feasibility.

Artificial intelligence systems require external validation in real training and competition environments. Researchers may test models across different disciplines, skill levels, ages, sexes, body types, equipment conditions, and recording environments. Comparisons with expert coaches, judges, biomechanical systems, and established clinical assessments would clarify their practical value.

Future studies may also evaluate the interpretability of automated outputs. Coaches and judges need to understand why a system identifies a technical error, predicts an injury risk, or recommends a specific correction. Research may determine which forms of feedback lead to better decisions and technical learning.

Randomised and controlled studies could compare traditional coaching feedback with immediate video feedback, wearable sensor feedback, and artificial intelligence-assisted instruction. Outcomes may include skill acquisition, retention, transfer, athlete understanding, dependence on external feedback, and safety.

Research on automated judging may examine measurable execution variables and artistic or contextual components separately. Studies may evaluate agreement with expert panels, consistency across competitions, resistance to recording errors, and procedures for disputed results. Athlete and judge acceptance may also receive attention.

Injury surveillance requires internationally consistent definitions of injury, exposure, severity, recurrence, and return to sport. Prospective registries may record the apparatus, technical element, training phase, workload, maturation status, previous injury, and mechanism associated with each condition.

Injury-prevention trials may evaluate multicomponent programmes that combine workload management, landing training, trunk control, strength, mobility, recovery, nutrition, and psychological support. Researchers may assess programme adherence and determine which components produce the greatest benefits for specific disciplines and age groups.

Long-term studies may follow gymnasts after retirement. Such research could clarify the relationship between competitive exposure, previous injuries, chronic pain, osteoarthritis, functional capacity, and quality of life. These data would help sports organisations balance performance objectives with long-term health.

Future research may include athletes in the design and evaluation of training and technological systems. Their perceptions of usability, trust, fatigue, pain, privacy, and feedback can identify practical problems that laboratory measurements may overlook.

Researchers must also address data protection, algorithmic bias, informed consent, and ownership of performance information. These issues require particular attention when technological systems collect health and movement data from children and adolescents.

## 5. Conclusions

This focused narrative review synthesized selected evidence identified through three predefined exact-phrase searches in the Web of Science Core Collection. Within this evidence corpus, the findings were organized around gymnastics training optimization, technological applications, and athlete health and injury prevention. The review provides an organizing perspective on these topics rather than a comprehensive representation of the multidisciplinary gymnastics literature or evidence for an established integrated training model.

Modern training methods may support the development of strength, power, coordination, mobility, movement control, and technical execution when appropriately adapted to the gymnast’s age, biological maturation, discipline, experience, and performance level. Progressive skill acquisition and carefully selected preparatory exercises can facilitate technical development while limiting unnecessary exposure to highly complex elements.

Technological applications, including video analysis, wearable sensors, motion capture, digital learning platforms, and artificial intelligence, offer promising opportunities for performance assessment, feedback, movement analysis, and technical evaluation. Their practical usefulness depends on considerations such as measurement validity, data reliability, transparent interpretation, and appropriate integration into coaching and judging. These technologies are therefore best considered as tools that complement professional expertise rather than replace specialist decision making.

The reviewed evidence also identifies a range of factors potentially relevant to athlete health and injury prevention. Biomechanical demands, landing control, training load, biological maturation, previous injuries, recovery, nutrition, menstrual health, sleep, and psychological status represent relevant factors for athlete monitoring. Early recognition of pain, fatigue, changes in technical execution, and rapid increases in training load may help inform timely adjustments to training and, when necessary, further medical assessment.

The findings also suggest potential value of a collaborative approach involving coaches, gymnasts, teachers, judges, sports scientists, medical professionals, psychologists, and families. Sharing relevant information among these stakeholders can help integrate technical development with workload management, recovery, athlete development, and health.

Interpretation of the included evidence requires caution because the focused search strategy may not have captured publications using alternative terminology or publications indexed outside the Web of Science Sport Sciences category. Additional limitations include methodological heterogeneity, small sample sizes, inconsistent injury definitions, and limited external validation of several technological systems. Importantly, the absence of a formal study-level methodological quality or risk-of-bias assessment means that studies with different levels of methodological robustness contributed to the narrative synthesis without formal quality-based weighting. Consequently, the findings may not be interpreted as definitive evidence of the effectiveness of specific interventions, and practical implications may be considered in light of this limitation. Future research may prioritize broader concept-based searches, standardized protocols, prospective and longitudinal designs, larger and more diverse samples, and validation under real training and competition conditions.

Overall, the reviewed evidence suggests that training decisions may benefit from considering physical preparation, technical development, athlete feedback, technological assessment, workload, recovery, and health monitoring together. However, this organizing perspective has not been tested as a complete intervention model. Its effectiveness, feasibility, and applicability across gymnastics disciplines require evaluation through prospective and controlled research.

## Figures and Tables

**Table 1:** Search strategy and results obtained from the Web of Science Core Collection.

Database	Exact Search Query	Period	Language	Principal WoS Category Used for Refinement	Records	Document-Type Filter
Web of Science Core Collection	TS = (“gymnastics training models”)	2005–2025	English	Sport Sciences	197	None
Web of Science Core Collection	TS = (“technology in gymnastics”)	2005–2025	English	Sport Sciences	194	None
Web of Science Core Collection	TS = (“injury prevention in gymnastics training”)	2005–2025	English	Sport Sciences	94	None
Total					485	

Note: The two books used exclusively for theoretical background were identified separately, were not retrieved through the database searches, and were not included among the 140 publications selected for the narrative synthesis. TS refers to the Topic field in the Web of Science Core Collection. Because the database is continuously updated, searches reproduced at a later date may retrieve a slightly different number of records. No document-type filter was applied during the database searches. Document types were assessed during screening according to the eligibility criteria described in Section 2.4. All searches were conducted in the Web of Science Core Collection using the Topic field and were restricted to the publication period from 1 January 2005 to 30 September 2025, English language, and the Sport Sciences category. No document-type filter was applied during the searches. No additional publications were identified through reference-list screening.

**Table 2:** Methodological evidence mapping of the included publications according to major review domain and principal study design.

Major Review Domain	Experimental/ Quasi- Experimental	Longitudinal	Cross-Sectional/ Observational	Biomechanical/ Validation	Qualitative	Review/ Conceptual	Total
Gymnastics training optimization	26	13	15	10	5	9	**78**
Technological applications in gymnastics	2	0	3	30	0	2	**37**
Athlete health and injury prevention	2	5	12	1	1	4	**25**
**Total**	**30**	**18**	**30**	**41**	**6**	**15**	**140**

Note: The table describes the methodological composition of the evidence base and should not be interpreted as a formal assessment of study quality, risk of bias, or certainty of evidence. Bold values indicate row and column totals.

**Table 3:** Chronological and thematic characteristics of the publications included in the narrative synthesis.

Characteristic	Category	Publications, n	Percentage, %
Publication period	2005 to 2019	54	38.6
	2020 to September 2025	86	61.4
Principal thematic area	Gymnastics training optimization	78	55.7
	Technological applications in gymnastics	37	26.4
	Athlete health and injury prevention	25	17.9

## Data Availability

No new data were created or analyzed in this study. Data sharing is not applicable to this article.

## Supplementary Materials

The following supporting information can be downloaded at: https://www.mdpi.com/article/10.3390/healthcare14193285/s1, Table S1: Characteristics of the publications included in the narrative synthesis.
